# Identifying older adults at risk of harm following elective surgery: a systematic review and meta-analysis

**DOI:** 10.1186/s12916-017-0986-2

**Published:** 2018-01-12

**Authors:** Jennifer Watt, Andrea C. Tricco, Catherine Talbot-Hamon, Ba’ Pham, Patricia Rios, Agnes Grudniewicz, Camilla Wong, Douglas Sinclair, Sharon E. Straus

**Affiliations:** 1 Division of Geriatric Medicine, University of Toronto, 27 King’s College Circle, Toronto, Ontario M5S 1A1 Canada; 2 Institute for Health Policy, Management and Evaluation, University of Toronto, 4th Floor, 155 College Street, Toronto, Ontario M5T 3M6 Canada; 3 Li Ka Shing Knowledge Institute, St. Michael’s Hospital, 209 Victoria Street, Toronto, Ontario M5B 1W8 Canada; 4 Epidemiology Division, Dalla Lana School of Public Health, University of Toronto, Health Sciences Building, 155 College Street, 6th floor, Toronto, Ontario M5T 3M7 Canada; 5 Toronto Health Economics and Technology Assessment Collaborative, Faculty of Pharmacy and Institute of Health Policy Management Evaluation, University of Toronto, 144 College Street, Toronto, Ontario M5S 3M2 Canada; 6 Telfer School of Management, University of Ottawa, 55 Laurier Avenue East, Ottawa, Ontario K1N 6N5 Canada

**Keywords:** Postoperative complications, Mortality, Functional decline, Elective surgery, Systematic review, Meta-analysis

## Abstract

**Background:**

Elective surgeries can be associated with significant harm to older adults. The present study aimed to identify the prognostic factors associated with the development of postoperative complications among older adults undergoing elective surgery.

**Methods:**

Medline, EMBASE, CINAHL, Cochrane Central Register of Controlled Trials, and AgeLine were searched for articles published between inception and April 21, 2016. Prospective studies reporting prognostic factors associated with postoperative complications (composite outcome of medical and surgical complications), functional decline, mortality, post-hospitalization discharge destination, and prolonged hospitalization among older adults undergoing elective surgery were included. Study characteristics and prognostic factors associated with the outcomes of interest were extracted independently by two reviewers. Random effects meta-analysis models were used to derive pooled effect estimates for prognostic factors and incidences of adverse outcomes.

**Results:**

Of the 5692 titles and abstracts that were screened for inclusion, 44 studies (12,281 patients) reported on the following adverse postoperative outcomes: postoperative complications (*n* =28), postoperative mortality (*n* = 11), length of hospitalization (*n* = 21), functional decline (*n* = 6), and destination at discharge from hospital (*n* = 13). The pooled incidence of postoperative complications was 25.17% (95% confidence interval (CI) 18.03–33.98%, number needed to follow = 4). The geriatric syndromes of frailty (odds ratio (OR) 2.16, 95% CI 1.29–3.62) and cognitive impairment (OR 2.01, 95% CI 1.44–2.81) were associated with developing postoperative complications; however, there was no association with traditionally assessed prognostic factors such as age (OR 1.07, 95% CI 1.00–1.14) or American Society of Anesthesiologists status (OR 2.62, 95% CI 0.78–8.79). Besides frailty, other potentially modifiable prognostic factors, including depressive symptoms (OR 1.77, 95% CI 1.22–2.56) and smoking (OR 2.43, 95% CI 1.32–4.46), were also associated with developing postoperative complications.

**Conclusion:**

Geriatric syndromes are important prognostic factors for postoperative complications. We identified potentially modifiable prognostic factors (e.g., frailty, depressive symptoms, and smoking) associated with developing postoperative complications that can be targeted preoperatively to optimize care.

**Electronic supplementary material:**

The online version of this article (doi:10.1186/s12916-017-0986-2) contains supplementary material, which is available to authorized users.

## Background

As the number of older adults increases globally, there will be a greater need for elective surgeries in this patient population; however, elective surgeries can be associated with significant harm to patients [1–5]. Special preoperative consideration must be given to the greater prevalence of geriatric syndromes faced by older adults, such as frailty and functional impairment, that potentially increase their risk of adverse postoperative outcomes [6, 7]. Indeed, older adults are a heterogeneous group of patients whose risk of adverse postoperative outcomes is not adequately described by chronological age, comorbidities, or the type of surgical procedure alone [8]. Although older adults are often seen in the preoperative medicine clinic for cardiovascular and respiratory risk stratification and optimization in anticipation of an elective surgery, little consideration is given to risk stratification for other adverse outcomes that occur in older adults, despite the availability of information to aid in this assessment [9].

Understanding the risk factors for postoperative complications may help clinicians, patients, and caregivers to target non-pharmacological and pharmacological interventions aimed at lessening the burden of these adverse postoperative outcomes. This systematic review synthesizes studies that identify preoperative prognostic factors of older adults undergoing elective surgery which may predispose them to adverse postoperative outcomes. This information can be used by clinicians and patients to enhance decision-making and management in the preoperative setting and by researchers to study possible interventions aimed at improving postoperative outcomes for older adults.

## Methods

This study was reported in accordance with both the PRISMA statement for reporting systematic reviews and meta-analyses and the MOOSE statement for reporting meta-analysis of observation studies in epidemiology (Additional file 1) [10, 11]. This systematic review and meta-analysis has a companion publication that focuses on prognostic factors associated with postoperative delirium among older adults undergoing elective surgery.

### Eligibility criteria

Prospective studies (e.g., randomized controlled trials (RCTs), quasi-RCTs, non-RCTs, controlled-before-and-after studies, prospective cohort studies) were eligible if they included older adults undergoing elective surgery (≥60 years old and mean age of patients enrolled in the study ≥ 65 years old) and reported prognostic factors associated with the postoperative complications of mortality, functional decline, prolonged length of hospitalization, discharge to a location other than home, and a composite outcome of medical or surgical complications. All definitions of a given prognostic factor were included. Studies that included patients ≥ 60 years old were selected to align with definitions from the United Nations and the World Health Organization [12, 13]. Geriatric medicine consultation services typically target these age ranges [14, 15]. Studies using any method for diagnosing postoperative complications were eligible. Postoperative mortality was defined as death within 30 days following surgery. If a study reported both elective and emergent surgical procedures, it was included in our systematic review only if there was a separate subgroup reported for patients undergoing elective surgery. To make the review feasible, studies reporting only clinical, laboratory, or imaging investigations that are not conducted as part of routine clinical practice (i.e., measuring serum interleukin levels) were excluded, as were studies disseminated in languages other than English.

### Information sources and search strategy

An experienced librarian searched MEDLINE (OVID interface, 1948 to April Week 3, 2016), EMBASE (OVID interface, 1980 to April Week 3, 2016), CINAHL (EBSCO interface, 1994 to April 21, 2016), Cochrane Central Register of Controlled Trials (Issue 4, April 2016), and AgeLine (EBSCO interface, 1968 to April 21, 2016) for potentially relevant studies. The full search strategy for MEDLINE (Additional file 2: Appendix 1) was modified as necessary for the other databases (full searches available upon request). Scanning the reference lists of included studies and searching the authors’ personal files supplemented the electronic search. Authors of conference proceedings were contacted to obtain unpublished work.

### Study selection

Two levels of screening were completed independently by two reviewers using *Synthesi*.SR (proprietary online software developed by the Knowledge Translation Program, Toronto, Canada); these were level 1 screening of titles and abstracts, and level 2, full-text screening of articles. A calibration exercise was conducted prior to level 1 screening whereby each reviewer independently screened 10% of a random sample of citations to ensure adequate inter-rater agreement. Study authors were contacted for further information if it was unclear whether the study met inclusion criteria. Disagreements concerning article inclusion were resolved through discussion; otherwise, a third reviewer was available to make a final decision.

### Data abstraction

Data were abstracted independently by two reviewers from studies retained from level 2 screening. Study characteristics (e.g., study design, country of conduct), patient characteristics (e.g., mean age, sex, comorbidities), and prognostic factors associated with the outcomes of interest were abstracted from included studies. Definitions operationalized by study authors for individual prognostic factors were also abstracted, where appropriate. Conflicts regarding the abstracted data were resolved through discussion. Authors were contacted for further information when the data were not clearly reported. The publication with the longest duration of follow-up was considered the major publication when multiple studies reported data from the same source. The other publications were retained as supplementary material only.

### Methodological quality assessment

Two reviewers independently appraised the risk of bias using the Cochrane Risk-of-Bias Tool for RCTs and the Newcastle–Ottawa Scale for cohort studies [16, 17]. We planned to assess other study designs with the Cochrane Effective Practice and Organization Care (EPOC) Risk-of-Bias Tool [18]. If two or more outcomes were reported in a single study, the quality assessment was preferentially conducted on the outcome of postoperative complications or destination at discharge from hospital.

### Statistical methods

We calculated odds ratios (OR) to quantify the relative risk of postoperative complications associated with each prognostic factor. Whenever only continuous effect measures, such as mean differences (e.g., age, body mass index) were reported, these effect sizes were transformed to OR estimates, if needed, to derive an overall effect estimate that combined both dichotomous and continuous study-level effect estimates [19]. For studies that reported multiple options with which to derive the study-level effect estimate (e.g., 2 × 2 tables, adjusted and unadjusted ORs, mean differences), the order of preference for selecting the source data is described in Additional file 2: Appendix 2.

Random effects models were used to derive overall effect estimates with 95% confidence intervals (CIs) when two or more studies reported extractable effect estimates that could be combined for the purpose of meta-analysis. The number needed to follow (NNF) was calculated as 1/pooled incidence of each postoperative complication. Similar to the concept of the number needed to treat or number needed to harm, the NNF represents the number of patients who need to be followed in a prognostic study in order to see one outcome [20]. Information regarding data imputation methods to approximate standard deviation values is found in Additional file 2: Appendix 3. Between-study statistical heterogeneity was quantitatively assessed with the I^2^ statistic and thresholds for the interpretation of the I^2^ statistic were consistent with those reported in the Cochrane Handbook for Systematic Reviews of Interventions [21].

Subgroup analyses were conducted by surgery type to explore between-study heterogeneity. Mixed-effects meta-regression models were also used to evaluate the effect of study-level effect modifiers (age, publication year, and type of surgery) on the pooled incidence of postoperative complications. Sensitivity analyses were conducted based on the type of study-level effect estimates used to calculate the overall effect estimates, - including study-level effect estimates that were adjusted for potentially important confounders only. A prognostic factor was considered significantly associated with the primary or secondary outcomes at a two-tailed *p-value* < 0.05. We planned to test for publication bias; however, this was not possible because there were no prognostic factors that were reported in at least 10 studies. All statistical analyses were conducted in R, version 3.2.4, using the *metafor* and *meta* packages [22, 23].

## Results

**Fig. 1:**
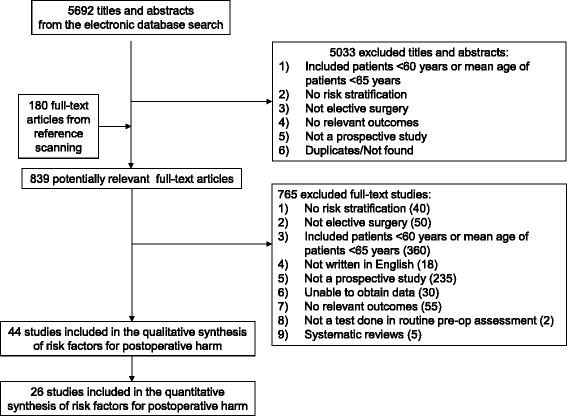
Study flow

Of the 5692 titles and abstracts that were screened for inclusion, 44 studies, including 12,281 patients, met our inclusion criteria (Fig. 1). From the 44 included studies, prognostic factors associated with postoperative complications (n = 28), postoperative mortality (n = 10), length of hospitalization (n = 22), functional decline (n = 6), and destination at discharge from hospital (n = 13) were retrieved. Two RCTs were included, which were at moderate to high risk of bias (Additional file 2: Appendix 4). Overall, the included cohort studies were of moderate to high methodological quality (Additional file 2: Appendix 5). The most common biases were the adequacy of follow-up of cohorts and the comparability of the cohorts on the basis of design.

### Postoperative complications

Twenty-eight studies (6708 patients) investigated the association between preoperative prognostic factors and postoperative complications (Additional file 1: Appendix 6). Of these, 23 were included in the meta-analyses of prognostic factors [1, 6, 7, 24–43]. The five studies not included in meta-analyses did not contain extractable data, report prognostic factors that were included in two or more studies, or present data in a format that could be pooled with other study-level effect estimates. Postoperative complications were most often reported as a composite of postoperative medical or surgical complications (e.g., pneumonia, wound infection, venous thromboembolism), the details of which are found in Additional file 2: Appendix 6. The pooled incidence of postoperative complications across all surgical types was 25.16% (95% CI 18.26–33.61%, 21 studies, I^2^ = 96%, NNF = 4) [1, 7, 25–28, 30–33, 35–38, 40–46]. In exploring the influence of type of surgery on incident complications, the number of complications remained high: cardiac surgery (9.46%, 95% CI 2.71–28.18%, 3 studies, I^2^ = 96.40%, NNF = 11), abdominal surgery (24.73%, 95% CI 8.63–53.33%, 3 studies, I^2^ = 96.1%, NNF = 5), and thoracic surgery (33.97%, 95% CI 12.66–64.62%, 4 studies, I^2^ = 95.5%, NNF = 3). The effects of the mean age of study patients, publication year, and type of surgery on the pooled incidence of postoperative complications were explored with meta-regression, but did not explain any of the variance in the models.

The prognostic factors most strongly associated with the development of postoperative complications were poor performance status as defined by the Eastern Cooperative Oncology Group (ECOG) score or the Karnofsky Index (OR 2.58, 95% CI 1.56–4.25, 5 studies, I^2^ = 0%), smoking status (OR 2.43, 95% CI 1.32–4.46, 3 studies, I^2^ = 0%), impairment in instrumental activities of daily living (IADLs) (OR 2.27, 95% CI 1.65–3.14, 6 studies, I^2^ = 0%), frailty (OR 2.16, 95% CI 1.29–3.62, 8 studies, I^2^ = 54.69%), and cognitive impairment (OR 2.01, 95% CI 1.44–2.81, 8 studies, I^2^ = 0%) (Table 1, Additional file 2: Appendix 7). Frailty was most frequently defined using the definition of Fried et al. [47]; however, other definitions included the Edmonton Frailty Scale, gait speed, or a tool created by individual study authors [48]. In a subgroup of frail patients undergoing abdominal surgery, there was no longer an association between frailty and postoperative complications (OR 1.73, 95% CI 0.81–3.66, 3 studies, I^2^ = 53.36%) [29, 35, 40]. These findings remained consistent when sensitivity analyses were conducted whereby only those studies reporting study-level effect estimates adjusted for important confounders were included.

**Table 1:** Prognostic factors for postoperative complications among older adults undergoing elective surgery

Prognostic factor	Number of studies	Number of patients	Odds ratio (95% CI)	Heterogeneity (I^2^)
Poor performance status	5	889	2.58 (1.56–4.25)	0
Smoking status	3	907	2.43 (1.32–4.46)	0
IADL impairment	7	1036	2.27 (1.65–3.14)	0
Frailty	8	1527	2.16 (1.29–3.62)	54.69
Cognitive impairment	8	1851	2.01 (1.44–2.81)	0
ADL impairment	4	829	1.98 (1.31–2.99)	0
Geriatric depression screen	4	777	1.77 (1.22–2.56)	0
Comorbidity score	5	1000	1.55 (1.29–1.87)	0
Depression	2	257	2.04 (0.67–6.23)	0
Poor mobility	2	477	2.51 (0.92–6.84)	63.37
Older age	9	2917	1.07 (1.00–1.14)	17.96
General anesthesia	2	172	0.78 (0.38–1.59)	0
ASA score ≥ 3	3	420	2.62 (0.78–8.79)	0
Malnutrition	7	847	1.22 (0.66–2.24)	31.02
Hypertension	3	912	0.90 (0.52–1.54)	0
Cerebrovascular disease	2	845	0.81 (0.11–5.94)	83.39
Diabetes mellitus	3	912	0.70 (0.39–1.26)	0
Polypharmacy	4	442	1.46 (0.9–2.37)	0
Male sex	6	2141	1.60 (0.88–2.91)	66.24

*ADL* activities of daily living, *ASA* American Society of Anesthesiologists, *IADL* instrumental activities of daily living, *CI* confidence interval

Other prognostic factors that were reported in single studies as significantly associated with postoperative complications were the cumulative number of impairments in the comprehensive geriatric assessment (OR 1.84, 95% CI 1.27–2.65), not being able to shop independently (*P* = 0.011), answering ‘yes’ to the question ‘Have you dropped many of your activities and interests?’ on the Geriatric Depression Scale (GDS) [49] (*P* = 0.04), the presence of one or more Goldman indicators [50] (*P* < 0.005), and the inability to bicycle 2 minutes to a heart rate greater than 99 beats/min (*P* < 0.05) [44, 45, 51]. The presence of anxiety (OR 5.1, 95% CI 1.27–20.2), Society of Thoracic Surgeons score [52] (OR 1.06, 95% CI 1.01–1.10), and female sex (OR 3.49, 95% CI 1.52–7.99) were associated with mortality or major morbidity in patients undergoing cardiac surgery [53].

Energy intake > 21.3 kcal/kg of actual body weight (OR 2.40, 95% CI 0.59–9.80), energy intake > 22.2 kcal/kg of ideal body weight (OR 5.00, 95% CI 0.95–26.17), or any of the items on the Nutrition Screening Initiative Nutritional Health Checklist [54] were not associated with postoperative complications [45, 46]. Besides answering ‘yes’ to the question ‘Have you dropped many of your activities and interests?’ on the GDS, none of the other questions were associated with postoperative complications. Similarly, no activities of daily living (ADLs) or IADLs, besides shopping, were individually associated with postoperative complications [45].

### Postoperative mortality

The association between preoperative prognostic factors and postoperative mortality was investigated in 11 studies (3399 patients) (Table 2) [1–3, 25, 30, 32, 51, 53, 55–57]. The pooled incidence of mortality was 4.58% (95% CI 3.67–5.71%, 11 studies, I^2^ = 46.30%, NNF = 21) [1–3, 25, 30, 32, 51, 53, 55–57]. Among patients undergoing cardiac surgery, the pooled incidence of mortality was 5.21% (4.00–6.75%, 6 studies, I^2^ = 60.8%, NNF = 20). Only the effects of publication year could be explored in a meta-regression because there were not enough studies to explore the effects of type of surgery or mean age of patients on the pooled incidence of mortality. Publication year did not explain any of the variance in the meta-regression model. Few prognostic factors were reported in more than one study. No significant association was identified between male sex (OR 1.46, 95% CI 0.67–3.19, 4 studies, I^2^ = 53.92%), diabetes mellitus (HR 1.74, 95% CI 0.54–5.61, 2 studies, I^2^ = 45.26%), or history of heart failure (HR 1.86, 95% CI 0.44–7.88, 2 studies, I^2^ = 68.34%) and postoperative mortality (Additional file 2: Appendix 8) [1, 30, 32, 55, 56].

**Table 2:** Prospective studies of risk factors for postoperative mortality among older adults undergoing elective surgery

Study	Number of patients	Number of deaths		Factors associated with postoperative mortality	Factors not associated with postoperative mortality
		*N*	%		
Audisio, 2008 [1]	460	16	3.5	Male sex (6.5% vs. 2.0%), more advanced cancer stage (*P* = 0.001)	Age (*P* > 0.05)
Badgwell, 2013 [2]	111	2	2	NR	No clinical, demographic, or CGA results were associated with morbidity or death
Betomvuko, 2015 [57]	94	4	4.2	Gait speed (0.68 ± 0.23 m/s vs. 0.43 ± 0.06 m/s, *P* = 0.037)	NR
Gerude, 2014 [30]	67	3	4.5	Male sex, IADL impairment	NR
Javierre, 2012 [32]	2038	74	3.6	Age (OR 2.28, 95% CI 1.52–3.43)	Male sex (OR 0.93, 95% CI 0.56–1.56)
Kim, 2013 [51]	141	6	4.3	NR	Cumulative number of impairments on CGA (OR 1.216, 95% CI 0.864–1.712, for death or post-discharge institutionalization)
Sundermann, 2014 [3]	455	28	6.1	CAF score (OR 1.1, 95% CI 1.06–1.12), FORECAST score (OR 1.3, 95% CI 1.2–1.5), EuroSCORE (OR 1.1, 95% CI 1.03–1.1), STS score (OR 1.3 (95% CI 1.1–1.5)	NR
Tamburino, 2011 [55]	663	39	5.9	Diabetes mellitus (HR 2.66, 95% CI 1.26–5.65), LVEF < 40% (HR 3.51, 95% CI 1.62–7.62)	EuroSCORE (c-statistic 0.55)
Wenaweser, 2011 [56]	200	15	7.5	BMI < 20 (HR 6.60, 95% CI 1.48–29.5), stroke (HR 4.41, 95% CI 1.16–16.8)	Age > 85 years (HR 1.69, 95% CI 0.17–16.5), male sex (HR 0.90, 95% CI 0.32–2.52), diabetes (HR 0.75, 95% CI 0.21–2.67), CHF (HR 0.79, 95% CI 0.25–2.47), COPD (HR 1.35, 95% CI 0.38–4.79), hypertension (HR 1.20, 95% CI 0.34–4.24), prior MI (HR 0.62, 95% CI 0.14–2.75), LVEF > 50% (HR 4.42, 95% CI 0.55–35.5), atrial fibrillation (HR 1.75, 95% CI 0.59–5.21)
Williams, 2013 [53]	148	7	4.7	NR	Anxiety (OR 2.53, 95% CI 0.26–24.82)

*BMI* body mass index, *CAF* comprehensive assessment of frailty, *CGA* comprehensive geriatric assessment, *CHF* congestive heart failure, *COPD* chronic obstructive pulmonary disease, *EuroSCORE* European System for Cardiac Operative Risk Evaluation, *FORECAST* Frailty predicts death one year after cardiac surgery test, *HR* hazard ratio, *IADL* instrumental activities of daily living, *LVEF* left ventricular ejection fraction, *MI* myocardial infarction, *STS* Society of Thoracic Surgeons, *CI* confidence interval, *N* number of patients, *NR* not reported, *OR* odds ratio

### Length of hospitalization

A total of 21 studies (5037 patients) investigated preoperative prognostic factors and length of hospitalization (Table 3) [1, 2, 7, 24, 25, 27, 29–31, 35–37, 39, 45, 46, 51, 53, 58–61]. Substantial between-study heterogeneity in the reporting of outcomes and few prognostic factors being reported in more than one study largely precluded pooling of study-level effect estimates. There was no association between higher American Society of Anesthesiologists (ASA) score and prolonged hospitalization (OR 0.82, 95% CI 0.30–2.23, 2 studies, I^2^ = 0%) (Additional file 2: Appendix 9) [2, 31]. Among six studies that investigated frailty as a prognostic factor for prolonged hospitalization, there was a significant association identified in four studies [7, 29, 35, 37, 40, 61]. One study found that, while frail patients with postoperative complications had prolonged hospitalizations (*P* < 0.001), those without postoperative complications did not (*P* = 0.19) [36]. Age was identified as a significant prognostic factor for prolonged hospitalization in only two of six studies [1, 2, 6, 26, 33, 51, 62–64].

**Table 3:** Prospective studies of risk factors associated with prolonged hospitalization among older adults undergoing elective surgery

Study	Number of patients	Overall length of hospitalization (days)		Factors associated with prolonged hospitalization	Factors not associated with prolonged hospitalization
		Median	IQR		
Audisio, 2008 [1]	460	5	3-70	Male sex (8 vs. 4 days, *P* < 0.005), more advanced cancer (2 vs. 11.5 days, *P* < 0.001), ADL dependent (RR 2.01, 95% CI 1.37–2.93), IADL dependent (RR 1.58, 95% CI 1.11–2.24), performance status abnormal (RR 1.64, 95% CI 1.06–2.56)	Age, MMSE abnormal (RR 1.18, 95% CI 0.76–1.86), GDS depressed (RR 1.30, 95% CI 0.91–1.85), ASA abnormal (RR 0.85, 95% CI 0.60–1.20), Satariano’s index (RR 1.23, 95% CI 0.85–1.78)
Badgwell, 2013 [2]	111	NR	NR	Cancer stage (distant vs. localized/regional) (OR 0.37, 95% CI 0.15–0.91), weight loss ≥ 10% (OR 4.03, 95% CI 1.13–14.43), polypharmacy (OR 2.45, 95% CI 1.09–5.48)	ASA score ≥ 2 (OR 1.02, 95% CI 0.39–2.69), ECOG performance status (OR 1.68, 95% CI 0.70–4.04)
Blakoe, 2015 [61]	79	NR	NR	NR	Frailty
Clement, 2011 [24]	1343	NR	NR	Age (Pearson’s coefficient 0.21, *P* < 0.001)	NR
Dasgupta, 2009 [7]	125	NR	NR	Age (*P* = 0.0074), Edmonton Frailty Scale score (*P* = 0.0042)	NR
Gerude, 2014 [30]	67	7^a^	2–26^b^	Smoking habit (OR 11.0, 95% CI 1.5–81.1), arm circumference ≤ 25 cm (OR 7.2, 95% CI 1.2–44.6), male sex (RR 2.15, 95% CI 1.10–4.18)^a^, IADL dependence (RR 1.97, 95% CI 1.07–3.61)^a^, underweight BMI (RR 2.23, 95% CI 1.19–4.55)^a^	Age > 78 years (RR 0.98, 95% CI 0.56–1.74), alcoholism (RR 0.84, 95% CI 0.47–1.48), ADL dependence (RR 0.94, 95% CI 0.47–1.89), advanced cancer stage (RR 1.64, 95% CI 0.60–4.49), type 2 diabetes mellitus (RR 2.28, 95% CI 0.63–8.13), hypertension (RR 1.21, 95% CI 0.63–8.13), COPD (RR 0.62, 95% CI 0.33–1.16), overweight BMI (RR 1.37, 95% CI 0.55–3.41), type 1 diabetes mellitus (RR 1.02, 95% CI 0.53–1.96), Karnofsky index ≤ 80 (RR 1.69, 95% CI 0.97–2.94), albumin ≤ 4.3 mg/dL (RR 1.10, 95% CI 0.63–2.02)
Green, 2012 [39]	159	NR	NR	Frailty (9 ± 6 days vs. 6 ± 5 days, *P* = 0.004)	NR
Huisman, 2014 [31]	280	NR	NR	TUG test > 20 s (OR 3.98, 95% CI 1.12–14.10), ASA score 2 (OR 0.23, 95% CI 0.05–0.99)	ASA score > 2 (OR 0.37, 95% CI 0.08–1.68)
Kenig, 2015 [36]	75	12	3–42	Frailty + postoperative complications (10.4 vs. 20.3 days, *P* < 0.001)	Frailty without postoperative complications (12.7 vs. 16.9 days, *P* = 0.19)
Kim, 2013 [51]	141	14	7–28	Cumulative number of impairments in CGA domains (*P* = 0.005)	NR
Kim, 2016 [37]	197	NR	NR	Mobility assessment tool – short form (HR 0.81, 95% CI 0.68–0.96), ASA status ≥ 3 (HR 1.65, 95% CI 1.11–2.46), intermediate- or high-risk surgery (HR 2.83, 95% CI 1.84–4.36), hs-CRP (HR 1.40, 95% CI 1.02–1.92)	Intermediately frail/frail (HR 1.13, 95% CI 0.79–1.60)
Kothari, 2011 [45]	60	NR	NR	Question 1 (6.55 ± 1.02 vs. 4.89 ± 0.84 days, *P* = 0.039), 9 (7.15 ± 1.16 vs. 5.04 ± 0.76 days, *P* = 0.01), and 10 (18.5 ± 9.50 vs. 5.04 ± 0.54 days, *P* = 0.031) of the NSI NHC	NR
Lasithoiotakis, 2013 [35]	57	NR	NR	Frailty (OR 4.2, 95% CI 1.3–13.5)	Age ≥ 75 years (OR 0.7, 95% CI 0.2–2.2), ASA score ≥ 3 (OR 5.6, 95% CI 0.8–35.8)
Makary, 2010 [29]	594	NR	NR	Frailty (IRR 1.69, 95% CI 1.28–2.23)	NR
Papaioannou, 2005 [27]	47	NR	NR	NR	General anesthesia (OR 2.50, 95% CI 0.74–8.46)
Reinohl, 2015 [59]	110	NR	NR	NR	Atrial fibrillation (*P* = 0.171), EuroScore I (*P* = 0.067), EuroScore II (*P* = 0.210), STS score (*P* = 0.220)
Robinson, 2012 [25]	186	12^a^	12^c^	Impaired cognition (15 ± 14 vs. 9 ± 9, *P* = 0.001)	NR
Rogers, 1989 [58]	46	14.5^a^	4.5^c^	Knee joint vs. hip (beta coefficient 4.3, standard error 1.3, *P* = 0.0021)	Osteoarthritis vs. rheumatoid arthritis (beta coefficient 1.3, SE 1.4, *P* = 0.3676)
Schmidt, 2015 [60]	652	9	7–14	TUG test > 21 s (regression coefficient 2.91, 95% CI 0.24–5.58, *P* = 0.03), malnutrition on MNA (regression coefficient 6.99, 95% CI 3.02–10.96, *P* = 0.001)	Above high school education (regression coefficient 0.18, 95% CI –0.79 to 1.15, *P* = 0.71), age (regression coefficient 0.08, 95% CI –0.23 to 0.19, *P* = 0.13), sex (regression coefficient 0.08, 95% CI –1.04 to 1.25, *P* = 0.86), tumor site (genitourinary vs. gastrointestinal) (regression coefficient –1.19, 95% CI –2.40 to 0.02, *P* = 0.05)
van Venrooij, 2009 [46]	100	NR	NR	NR	Protein intake > 0.98 g/kg actual body weight (OR 0.857, 95% CI 0.376–1.956), protein intake > 0.98 g/kg ideal body weight (OR 0.857, 95% CI 0.376–1.956), energy intake > 21.3 kcal/kg actual body weight (OR 1.221, 95% CI 0.535–2.786), energy intake > 22.2 kcal/kg ideal body weight (OR 1.221, 95% CI 0.535–2.786)
Williams, 2013 [53]	148	NR	NR	NR	Anxiety vs. none (OR 1.20, 95% CI 0.30–4.87)

^a^Mean

^b^Range

^c^Standard deviation

*ADLs* activities of daily living, *ASA* American Society of Anesthesiologists, *BMI* body mass index, *COPD* chronic obstructive pulmonary disease, *ECOG* Eastern Cooperative Oncology Group, *GDS* geriatric depression scale, *hs-CRP* high sensitivity C-reactive protein, *IADLs* instrumental activities of daily living, *MMSE* Mini Mental State Exam, *MNA* Mini Nutritional Assessment, *NSI NHC* Nutrition Screening Initiative Nutritional Health Checklist, *TUG* timed up and go, *CI* confidence interval, *HR* hazard ratio, *IQR* interquartile range, *NR* not reported, *OR* odds ratio, *RR* relative risk

### Destination at discharge from hospital

In total, 13 studies (2601 patients) investigated associations between preoperative prognostic factors and the destination at discharge from hospital (e.g., skilled nursing facility vs. discharge to home) (Table 4) [2, 7, 25, 29, 37, 38, 40, 45, 51, 53, 61, 65, 66]. Patients were discharged to a number of locations, including other hospitals, nursing homes, rehabilitation centres, transitional care facilities, and assisted-living facilities. The pooled incidence of discharge from hospital to a destination other than home was 13.65% (8.90–20.39%, 9 studies, I^2^ = 91.6%, NNF = 8) [2, 25, 37, 38, 40, 45, 51, 53, 66]. In a subgroup of older adults undergoing general surgery, the pooled incidence of being discharged to a non-home location was 9.97% (6.59–12.39%, 2 studies, I^2^ = 0%, NNF = 11) [2, 40]. Only the effects of publication year could be explored in a meta-regression because there were not enough studies to explore the effects of type of surgery or mean age of patients on the pooled incidence of mortality. Publication year did not explain any of the variance in the meta-regression model. In another subgroup of older adults, the pooled incidence of being discharged to a nursing home was 9.97% (5.30–17.96%, 2 studies, I^2^ = 86%) [37, 66]. Meta-analysis of data from five studies (1228 patients) found that frailty was associated with non-home discharge following elective surgery (OR 3.42, 95% CI 1.35–8.68, I^2^ = 67.46%) (Additional file 2: Appendix 10) [7, 29, 37, 38, 40]. In an additional study, the odds of being transferred to another hospital were six times greater for frail patients (*P* = 0.002) (Table 4) [61]. There were a number of prognostic factors that were associated with an increased risk of non-home destination at discharge from hospital, namely older age, weight loss ≥ 10%, ASA score ≥ 2, ECOG performance status ≥ 2, and lower self-reported mobility [2, 7, 37, 66, 67].

**Table 4:** Prospective studies of risk factors associated with non-home discharge among older adults undergoing elective surgery

Study	Number of patients	Non-home discharge		Discharge destination	Factors associated with non-home discharge	Factors not associated with non-home discharge
		N	%			
Badgwell, 2013 [2]	111	11	10	Skilled nursing facility (including inpatient rehabilitation facilities)	Weight loss ≥ 10% (OR 6.52, 95% CI 1.43–29.76), ASA score ≥ 2 (OR 5.08, 95% CI 1.13–22.78), ECOG performance status ≥ 2 (OR 4.51, 95% CI 1.03–19.71)	Polypharmacy (OR 1.33, 95% CI 0.38–4.64), distant stage cancer (OR 0.54, 95% CI 0.11–2.64)
Blakoe, 2015 [61]	79	NR	NR	Another hospital	Frailty (OR 6, *P* = 0.002)	NR
Courtney-Brooks, 2012 [38]	37	1	2.7	Skilled nursing facility	NR	Frailty (*P* = 0.25)
Dasgupta, 2009 [7]	125	NR	NR	Institution	Age (*P* = 0.0009), Edmonton Frailty Scale score (*P* = 0.013)	NR
Kim, 2013 [51]	141	26	19.3	Nursing home, transitional care facility, or acute care facility	NR	Cumulative number of impairments on CGA (OR 1.216, 95% CI 0.864–1.712, for death or post-discharge institutionalization)
Kim, 2014 [40]	275	24	8.7	Nursing home, transitional care, or any long-term care facility	Frailty (OR 1.42, 95% CI 1.09–1.86)	NR
Kim, 2016 [37]	197	27	13.7	Nursing home	Mobility assessment tool – short form (OR 2.01, 95% CI 1.13–3.56), intermediately frail/frail (OR 3.11, 95% CI 1.02–9.54), age (OR 1.15, 95% CI 1.05–1.27), preoperative pain score (OR 0.83, 95% CI 0.70–0.99)	NR
Kothari, 2011 [45]	60	6	10	Location other than home	IADL score for ‘shopping’ (*P* = 0.003)	IADL scores (food preparation, housekeeping, laundry, medications, managing money, telephone usage, transportation), NSI Nutritional Health Checklist, GDS
Legner, 2004 [66]	586	43	14	Nursing home	Age 70–74 years vs. < 65 years (OR 5.4, 95% CI 1.9–15.7), 75–79 years (OR 10.5, 95% CI 3.7–29.5), ≥ 80 years (OR 16.3, 95% CI 5.5–48.7)	Age 65–69 years vs. < 65 years (OR 2.5, 95% CI 0.8–8.3)
Makary, 2010 [29]	594	NR	NR	Skilled or assisted-living facility	Frailty (OR 20.48, 95% CI 5.54–75.68)	NR
Min, 2015 [65]	49	NR	NR	All non-home locations	NR	Any of the baseline geriatric assessments
Robinson, 2012 [25]	186	52	29	Institutional care facility (i.e., nursing home, skilled nursing facility or rehabilitation centre)	Impaired cognition (OR 3.01, 95% CI 1.55–5.86)	NR
Williams, 2013 [53]	148	47	31.8	Healthcare facility	NR	Anxiety vs. none (OR 2.29, 95% CI 0.65–8.10)

*ASA* American Society of Anesthesiologists, *CGA* comprehensive geriatric assessment, *ECOG* Eastern Cooperative Oncology Group, *GDS* geriatric depression scale, *IADL* instrumental activities of daily living, *NSI* Nutrition Screening Initiative, *CI* confidence interval, *NR* not reported, *OR* odds ratio

### Functional decline

Six studies (1426 patients) investigated the association between preoperative prognostic factors and postoperative functional decline (Table 5) [63–65, 68–70]. All six studies reported prognostic factors associated with postoperative impairment in a patient’s ability to perform ADLs. One study reported risk factors associated with postoperative impairment in the ability to perform IADLs [63]. The pooled incidence of decline in ADLs was 21.03% (9.94–39.11%, 4 studies, I^2^ = 97.1%, NNF = 5) [63, 64, 69, 70]. In a subgroup of patients undergoing general surgery, the pooled incidence of decline in ADLs was 15.25% (5.48–35.83%, 2 studies, I^2^ = 95.7%, NNF = 7) [63, 64]. Age was not found to be associated with postoperative impairment in ADLs at 4–6 weeks, 3 months, or 1 year after elective surgery; however, one study did show an association between age and impairment in ADLs in the postoperative period [63–65, 68, 70]. Baseline MMSE score was not associated with a decline in ADLs, but it was associated with a decline in IADLs [63, 64].

**Table 5:** Prospective studies of risk factors associated with functional decline among older adults undergoing elective surgery

Study	Number of patients	Number of patients with functional decline		Time point(s) functional decline measured	Prognostic factors associated with functional decline	Prognostic factors not associated with functional decline
		N	%			
Amemiya, 2007 [64]	223	NR	24	1, 3, and 6 months	POSSUM model (OR 1.19, 95% CI 1.05–1.34), E–PASS model (OR 1.26, 95% CI 1.08–1.47), age (OR 1.10, 95% CI 1.03–1.17)	APACHE II model (OR 1.08, 95% CI 0.97–1.20), male sex (OR 1.72, 95% CI 0.95–3.12), MMSE score (OR 0.95, 95% CI 0.87–1.03), colon cancer (OR 0.89, 95% CI 0.52–1.51)
Hoogerduijn, 2014 [69]	475	74	16	3 months	ISAR-HP: ≥ 65 years (AUC 0.72, 95% CI 0.65–0.79), ≥ 70 years (AUC 0.73, 95% CI 0.66–0.80), ≥ 75 years (AUC 0.75, 95% CI 0.66–0.83)	NR
Kwon, 2012 [70]	204	93	45.3	1, 3, and 12 months	Male sex (OR 3.05, 95% CI 1.41–6.58) at 1 month, ASA score (OR 3.41, 95% CI 1.31–8.86) at 3 months, cancer (OR 2.6, 95% CI 1.14–5.96) at 12 months, smoking status (OR 3.15, 95% CI 1.27–7.85) at 3 months	Age (OR 1.07, 95% CI 0.99–1.15, at 1 month; non-significant all time points), male sex (OR 2.23, 95% CI 0.98–5.08, at 3 months) and (OR 1.17, 95% CI 0.49–2.78, at 12 months), ASA score (OR 1.29, 95% CI 0.58–2.87, at 1 month) and (OR 1.41, 95% CI 0.59–3.34, at 12 months), depression (OR 2.04, 95% CI 0.95–4.36, at 1 month; non-significant all time points), cancer (OR 1.52, 95% CI 0.68–3.39, at 1 month) and (OR 1.29, 95% CI 0.58–2.89, at 3 months), smoking status (OR 1.67, 95% CI 0.79–3.53, at 1 month) and (OR 1.04, 95% CI 0.46–2.36, at 12 months), physical function score – middle tertile (OR 0.68, 95% CI 0.28–1.66, at 1 month; non-significant all time points)
Lawrence, 2004 [63]	372	55	17	ADL recovery at 3 months, IADL recovery at 6 months	Physical status (OR 1.42, 95% CI 1.06–1.90, ADL recovery) and (OR 1.45, 95% CI 1.04–2.03, IADL recovery), MMSE score (OR 1.18, 95% CI 1.02–1.34, IADL recovery), GDS (OR 0.91, 95% CI 0.85–0.98, IADL recovery), creatinine > 1.5 mg/dL (OR 0.21, 95% CI 0.06–0.70, IADL recovery), albumin < 3 mg/dL (OR 0.11, 95% CI 0.01–1.22, IADL recovery)	Social support (OR 1.01, 95% CI 1.00–1.02, ADL recovery), MMSE score (OR 1.04, 95% CI 0.92–1.18, ADL recovery), IADL performance (OR 0.96, 95% CI 0.83–1.10, ADL recovery), age (OR 0.97, 95% CI 0.92–1.02, ADL recovery) and (OR 0.99, 95% CI 0.94–1.06, IADL recovery), male sex (OR 0.95, 95% CI 0.38–2.37, IADL recovery), age (OR 0.99, 95% CI 0.94–1.06, IADL recovery)
Min, 2015 [65]	49	NR	NR	4–6 weeks	Baseline ADL impairment (*P* < 0.05), comorbidity (*P* = 0.03)	Age
Pirracchio, 2010 [68]	90	NR	NR	12 months	ADL score at admission (OR 1.67, 95% CI 1.10–2.54), meningioma (vs. others) (OR 3.92, 95% CI 1.43–10.73)	Age (OR 0.95, 95% CI 0.85–1.07), sex (OR 0.83, 95% CI 0.35–1.94), GDS (OR 1.13, 95% CI 0.52–2.45), ASA score (OR 0.55, 95% CI 0.24–1.26), KPS score (OR 1.03, 95% CI 1.03, 95% CI 1.00–1.06), focal deficit (OR 0.52, 95% CI 0.20–1.32)

*ADLs* activities of daily living, *APACHE II* Acute Physiology and Chronic Health Evaluation, *ASA* American Society of Anesthesiologists, *E-PASS* Estimation of Physical Ability and Surgical Stress, *GDS* Geriatric Depression Scale, *IADLs* instrumental activities of daily living, *ISAR-HP* Identification of Seniors at Risk-Hospitalized Patients, *KPS* Karnofsky performance status, *MMSE* Mini Mental State Exam, *POSSUM* Physiological and Operative Severity Scoring System, *CI* confidence interval, *AUC* area under the curve, *N* number of patients, *OR* odds ratio

## Discussion

This systematic review and meta-analysis identified preoperative prognostic factors associated with the risk of harm in older adults undergoing elective surgery. Common geriatric syndromes, such as functional impairment, cognitive impairment, and frailty, were associated with the composite outcome of postoperative complications, while more traditional perioperative risk factors in the medical literature, such as older age and ASA status, were not [71]. Although the pooled incidences of adverse postoperative outcomes must be interpreted with caution because of significant between-study heterogeneity, it is worth noting that approximately one in four older adults suffered a postoperative complication from undergoing elective surgery. Fortunately, we identified a number of potentially modifiable risk factors, including smoking status, depressive symptoms, and frailty, that can be explored in future studies aimed at preventing adverse postoperative outcomes in older adults undergoing elective surgery.

The finding that geriatric syndromes, but not older age or ASA status, were associated with postoperative complications warrants further discussion. In particular, frailty is felt to represent a patient’s biological age as opposed to their chronological age, which may explain why frailty and not older age was associated with postoperative complications in this setting [72]. Frail patients were also less likely to be discharged to their home, which again likely reflects their decreased physiological reserve to respond to a significant stressor such as surgery. Besides being associated with postoperative complications, frailty has been associated with a number of other adverse outcomes outside of the perioperative literature, including mortality and admission to a long-term care facility [73, 74]. Perhaps greater emphasis should be placed on a patient’s frailty status as opposed to their age in determining risk of adverse postoperative outcomes as part of a comprehensive preoperative assessment [9].

The high incidence of adverse outcomes (25% of patients experiencing a postoperative complication), even in this non-emergent surgical setting, was also surprising. There was significant between-study heterogeneity among studies reporting postoperative complications, which could not be completely explained by type of surgery, but instead likely reflects the range of postoperative complications that were reported by study authors (e.g., atelectasis, venous thromboembolism, death). In the future, it will be important for more researchers to identify postoperative complications by severity so that knowledge users (e.g., patients, clinicians) can have a better-informed discussion as to a patient’s risk of developing different postoperative complications.

To our knowledge, this is the first systematic review and meta-analysis that comprehensively examined the association between preoperative prognostic factors and adverse postoperative outcomes among older adults undergoing elective surgery. A recent narrative review on adverse postoperative outcomes among older adults included patients with different indications for surgery, such as hip fracture or other emergent procedures, and did not conduct meta-analyses of prognostic factors [75]. We targeted older adults undergoing elective surgery because of the potential to intervene to improve patient outcomes by identifying and optimizing these factors preoperatively. Multicomponent interventions aimed at improving a patient’s nutrition, physical fitness, and cognition have shown promise in improving frailty [76]. Similarly, smoking status and depressive symptoms are potentially modifiable prognostic factors that were associated with developing postoperative complications. Interventions for preoperative smoking cessation have been associated with a lower risk of postoperative complications [77]. These prognostic factors could be targeted in the preoperative clinic.

There were limitations in our study’s review process. Firstly, only studies that were published in English were included in this review to increase feasibility, but our findings are likely generalizable given the number of geographical regions represented in our systematic review. Secondly, there was substantial heterogeneity between studies for some outcomes, which could not always be adequately explored given a limited number of studies and a lack of individual patient-level data. Indeed, it is possible that by including such a broad spectrum of elective surgical procedures we may create difficulty in understanding exactly which prognostic factors are most likely to be important for certain patients, but this was explored in subgroup analyses and meta-regression models, where possible. Additionally, this study was initiated prior to the introduction of the CHARMS checklist, which means that biases introduced in model development, validation, and evaluation of our included studies are less well described; however, we feel that we were able to identify important sources of selection bias, measurement bias, and confounding that threatened the validity of individual study findings [78].

There were also limitations imparted by the included studies themselves. The methodological quality assessment demonstrated that there were a number of studies reporting varying intensity of follow-up, which may have impacted the incidence of complications. The majority of studies included in this systematic review were cohort studies; therefore, our findings may be influenced by confounding. Sensitivity analyses demonstrated that our findings were largely consistent when only study-level effect estimates that were adjusted for potentially important confounders were included in the meta-analyses. Lastly, sometimes studies did not report independent variables for which there was a non-significant association with the dependent variable in the final multivariable model, which could potentially lead to a type 1 error in the findings of our meta-analyses. This is a limitation that is inherent in the prognosis literature that we hope will be overcome in the future by improved quality of reporting.

Our study had a number of strengths. There were 44 studies and over 12,000 patients included in our systematic review and meta-analyses, which allowed us to investigate a number of possible prognostic factors. The hypothesis-generating nature of this study allowed for the identification of prognostic factors that are potentially modifiable in the preoperative setting, which could lead to better surgical outcomes for older adults undergoing elective surgery.

## Conclusions

In summary, this systematic review and meta-analysis highlights how common postoperative complications are among older adults undergoing elective surgery (NNF = 4) and the importance of geriatric syndromes in identifying older adults at risk of harm. Furthermore, there were several prognostic factors identified that could be modifiable in a preoperative setting, including smoking and frailty, which can be explored in future knowledge translation strategies to develop interventions aimed at mitigating the risk faced by older adults undergoing elective surgery.

## Additional files

Additional file 1: PRISMA Checklist. (DOCX 30 kb)

Additional file 2: Appendix 1. MEDLINE search strategy. **Appendix 2.** Order preference for combining data types in meta-analyses. **Appendix 3.** Data imputation methods. **Appendix 4.** Cochrane risk of bias assessment for randomized trials. **Appendix 5.** Newcastle–Ottawa scale for evaluating the quality of cohort studies. **Appendix 6.** Table of characteristics of prospective studies reporting prognostic factors associated with postoperative complications among older adults undergoing elective surgery. **Appendix 7.** Forest plots of study-level and pooled effect estimates for prognostic factors associated with postoperative complications among older adults undergoing elective surgery. **Appendix 8.** Forest plots of the study-level and pooled effect estimates of the prognostic factors associated with postoperative mortality among older adults undergoing elective surgery. **Appendix 9.** Forest plot of study-level and pooled effect estimates for prognostic factors associated with prolonged hospitalization among older adults undergoing elective surgery. **Appendix 10.** Forest plot of study-level and pooled effect estimates for prognostic factors associated with destination at discharge from hospital among older adults undergoing elective surgery (DOCX 550 kb)

## Abbreviations

ADL: activities of daily living
ASA: American Society of Anesthesiologists
CI: confidence interval
ECOG: Eastern Cooperative Oncology Group
GDS: geriatric depression scale
HR: hazard ratio
IADL: instrumental activities of daily living
IQR: interquartile range
MMSE: Mini Mental State Exam
NNF: number needed to follow
OR: odds ratio
RCT: randomized controlled trial
RR: relative risk.
