# Supplementary material for: Identifying older adults at risk of harm following elective surgery: a systematic review and meta-analysis
Source: BMC Med. 2018 Jan 12;16:2. doi: 10.1186/s12916-017-0986-2 (PMC5765656; doi:10.1186/s12916-017-0986-2)
Supplement: Supplementary file 2 — MEDLINE search strategy. Appendix 2. Order preference for combining data types in meta-analyses. Appendix 3. Data imputation methods. Appendix 4. Cochrane risk of bias assessment for randomized trials. Appendix 5. Newcastle–Ottawa scale for evaluating the quality of cohort studies. Appendix 6. Table of characteristics of prospective studies reporting prognostic factors associated with postoperative complications among older adults undergoing elective surgery. Appendix 7. Forest plots of study-level and pooled effect estimates for prognostic factors associated with postoperative complications among older adults undergoing elective surgery. Appendix 8. Forest plots of the study-level and pooled effect estimates of the prognostic factors associated with postoperative mortality among older adults undergoing elective surgery. Appendix 9. Forest plot of study-level and pooled effect estimates for prognostic factors associated with prolonged hospitalization among older adults undergoing elective surgery. Appendix 10. Forest plot of study-level and pooled effect estimates for prognostic factors associated with destination at discharge from hospital among older adults undergoing elective surgery (DOCX 550 kb) [file 12916_2017_986_MOESM2_ESM.docx]

**Additional file 1**

Supplementary Online Content

[Appendix 1. MEDLINE search strategy 2](#_Toc493140540)

[Appendix 2. Order preference for combining data types in meta-analyses 3](#_Toc493140541)

[Appendix 3. Data imputation methods 4](#_Toc493140542)

[Appendix 4. Cochrane risk of bias assessment for randomized trials 5](#_Toc493140543)

[Appendix 5. Newcastle-Ottawa scale for evaluating the quality of cohort studies 6](#_Toc493140544)

[Appendix 6. Table of characteristics of prospective studies reporting prognostic factors associated with postoperative complications among older adults undergoing elective surgery 10](#_Toc493140545)

[Appendix 7. Forest plots of study-level and pooled effect estimates for prognostic factors associated with postoperative complications among older adults undergoing elective surgery 15](#_Toc493140546)

[Appendix 8. Forest plots of the study-level and pooled effect estimates of the prognostic factors associated with postoperative mortality among older adults undergoing elective surgery 36](#_Toc493140547)

[Appendix 9. Forest plot of study-level and pooled effect estimates for prognostic factors associated with prolonged hospitalization among older adults undergoing elective surgery 39](#_Toc493140548)

[Appendix 10. Forest plot of study-level and pooled effect estimates for prognostic factors associated with destination at discharge from hospital among older adults undergoing elective surgery 40](#_Toc493140549)

[References 41](#_Toc493140550)

# Appendix 1. MEDLINE search strategy

Database: Ovid MEDLINE(R) <1948 to April Week 23 2016>, Ovid MEDLINE(R) In-Process & Other Non-Indexed Citations <April 21, 2016> Search Strategy:
--------------------------------------------------------------------------------
1 (perioperative or peri-operative).mp.
2 (preoperative or pre-operative).mp.
3 (preadmission or pre-admission).mp.
4 or/1-3
5 (geriatrics or geriatric).mp.
6 (elderly or senior? or (old adj age) or (older adj adult?)).mp.
7 Health Services for the Aged/
8 or/5-7
9 Geriatric Assessment/
10 assessment?.mp.
11 or/9-10
12 4 and 8 and 11
13 (animals not (humans and animals)).sh.
14 12 not 13

# Appendix 2. Order preference for combining data types in meta-analyses

| **Type of Data** | **Example of Data Types** | **Pooling Preference Across Dichotomized Data** | **Pooling Preference Across All Data Types** |
| --- | --- | --- | --- |
| Raw Data | Sex, Presence of Absence of a Medical Disease | 2 | 3 |
| Unadjusted Odds Ratio | Sex, Presence of Absence of a Medical Disease | 3 | 4 |
| Adjusted Odds Ratio | Categorical or Continuous Data as Reported in a Multivariable Regression Model | 1 | 1 |
| Mean Difference | Age, Body Mass Index | Not applicable | 2 |
| Standardized Mean Difference | Combining Different Scales for Cognitive Impairment | Not applicable | 2 |

**Note:** Data reported as a mean difference or standardized mean difference was transformed into an odds ratio using the method of Sanchez-Meca et al., 2003, in the metafor package [1].

# Appendix 3. Data imputation methods

If only 95% confidence intervals were available to represent the variance of a mean value, the standard deviation was estimated as per the following equation [2]:

*Standard deviation = √number of patients in the group x (upper bound of the confidence interval – lower bound of the confidence interval)/(appropriate value from t-distribution x 2)*

Similar imputations were not conducted for hospital length of stay because the data distribution was likely to be right-skewed.

# Appendix 4. Cochrane risk of bias assessment for randomized trials

| **Author, Year** | **Random sequence generation** | **Allocation concealment** | **Blinding of participants and personnel** | **Blinding of outcome assessment** | **Incomplete outcome data** | **Selective reporting** | **Other bias** |
| --- | --- | --- | --- | --- | --- | --- | --- |
| Papaioannou, 2005[3] | Low Risk | Unclear Risk | Unclear Risk | Unclear Risk | Low Risk | Low Risk | High Risk |
| Schmidt, 2015[4] | Low Risk | Unclear Risk | High Risk | High Risk | Low Risk | Low Risk | High Risk |

# Appendix 5. Newcastle-Ottawa scale for evaluating the quality of cohort studies

| **Author, Year** | **Representative exposed cohort** | **Selection of the non- exposed cohort** | **Ascertainment of exposure** | **Demonstration that outcome of interest was not present at start of study** | **Comparability of cohorts on the basis of the design or analysis** | **Assessment of outcome** | **Follow-up long enough for outcomes to occur** | **Adequacy of follow up of cohorts** |
| --- | --- | --- | --- | --- | --- | --- | --- | --- |
| Amemiya, 2007[5] | * | * | * | * | ** | * | * | * |
| Audisio, 2008[6] | * | * | * | * | ** | * | * |  |
| Aykut, 2013[7] | * | * | * | * |  | * |  |  |
| Badgwell, 2013[8] | * | * | * | * | * | * | * | * |
| Betomvuko, 2015[9] | * | * | * | * |  | * | * |  |
| Blakoe, 2015[10] | * | * | * |  |  | * |  | * |
| Clement, 2011[11] | * | * | * |  |  |  |  |  |
| Courtney-Brooks, 2012[12] | * | * | * | * |  | * | * |  |
| Dales, 1993[13] | * | * | * | * |  | * |  |  |
| Dasgupta, 2009[14] | * | * | * |  | * | * |  |  |
| Fukuse, 2005[15] | * | * | * |  | * |  | * |  |
| Gerson, 1985[16] | * | * | * | * | * | * |  |  |
| Gerude, 2014[17] | * | * | * | * | * | * | * |  |
| Goto, 2007[18] | * | * | * | * | * | * |  | * |
| Green, 2012[19] | * | * | * | * | ** | * | * |  |
| Hoogerduijn, 2014[20] | * | * | * | * | * | * | * | * |
| Huisman, 2014[21] | * | * | * | * | * | * | * |  |
| Javierre, 2012[22] | * | * | * | * |  | * |  |  |
| Kenig, 2015[23] | * | * | * | * | ** | * | * | * |
| Kim, 2013[24] | * | * | * |  | * | * |  |  |
| Kim, 2014[25] | * | * | * | * | ** | * |  | * |
| Kim, 2016[26] | * | * | * | * | ** | * | * | * |
| Kothari, 2011[27] | * | * | * | * |  | * | * | * |
| Kristjansson, 2010[28] | * | * | * | * | ** | * | * | * |
| Kwon, 2012[29] | * | * | * | * | ** | * | * | * |
| Lasithiotakis, 2013[30] | * | * | * | * |  | * | * |  |
| Lawrence, 2004[31] | * | * | * | * | ** | * | * | * |
| Legner, 2004[32] | * | * | * | * | ** |  | * |  |
| Makary, 2010[33] | * | * | * | * | * | * | * |  |
| Min, 2015[34] | * | * | * | * | * | * | * | * |
| Pirracchio, 2010[35] | * | * | * | * | * | * | * |  |
| Reinohl, 2015[36] | * | * | * | * | * | * | * |  |
| Robinson, 2012[37] | * | * | * |  |  | * |  |  |
| Rogers, 1989[38] | * | * | * |  | ** | * | * | * |
| Suh, 2014[39] | * | * | * |  | ** | * | * | * |
| Sundermann, 2014[40] | * | * | * | * | * | * | * |  |
| Tamburino, 2011[41] | * | * | * | * | * | * | * | * |
| Tan, 2012[42] | * | * | * | * |  | * | * |  |
| van Venrooij, 2009[43] | * | * | * | * | * | * | * |  |
| Wenaweser, 2011[44] | * | * | * | * | ** | * | * | * |
| Williams, 2013[45] | * | * | * | * | ** | * |  | * |
| Zhang, 2012[46] | * | * | * | * |  | * |  |  |

**Newcastle-Ottawa scale for evaluating the quality of cohort studies**

*Note: A study can be awarded a maximum of one star for each numbered item within the Selection and Outcome categories. A maximum of two stars can be given for Comparability.*

**Selection**

1. Representativeness of the exposed cohort

a) truly representative of the average older adult in the community *****

b) somewhat representative of the average older adult in the community *****

c) selected group of users (e.g., nurses, volunteers)

d) no description of the derivation of the cohort

2. Selection of the non-exposed cohort

a) drawn from the same community as the exposed cohort *****

b) drawn from a different source

c) no description of the derivation of the non-exposed cohort

3. Ascertainment of exposure

a) secure record (e.g., surgical records) *****

b) structured interview *****

c) written self-report

d) no description

4. Demonstration that outcome of interest was not present at start of study

a) yes *****

b) no

**Comparability**

1. Comparability of cohorts on the basis of the design or analysis

a) study controls for age*

b) study controls for any additional factor (e.g., cognitive impairment, baseline comorbidities)*****

**Outcome**

1. Assessment of outcome

a) independent blind assessment*

b) record linkage*

c) self-report

d) no description

2. Was follow-up long enough for outcomes to occur

a) yes (follow-up ≥2 days)*

b) no

3. Adequacy of follow up of cohorts

a) complete follow up - all subjects accounted for*

b) subjects lost to follow up unlikely to introduce bias - small number lost - < 10% or description provided of those lost*

c) follow up rate <90% and no description of those lost

d) no statement

# Appendix 6. Table of characteristics of prospective studies reporting prognostic factors associated with postoperative complications among older adults undergoing elective surgery

| **Study** | **Number of Patients** | **Age Range (years)** | **% Female** | **Type of Surgery** | **Exclusion Criteria at Baseline** | **Number of Patients with Complications** | | **Complication(s)** |
| --- | --- | --- | --- | --- | --- | --- | --- | --- |
|  |  |  |  |  |  |  |  |  |
|  |  |  |  |  |  | **N** | **%** |  |
| Audisio, 2008[6] | 460 | 70-95 | 65.9 | Breast, gastro-intestinal, genitourinary, other | MMSE <18 | 171 | 37.8 | Wound infection, respiratory morbidity, nutritional problem, other |
| Aykut, 2013[7] | 48 | >70 | 54.2 | Cardiac | Severe cognitive impairment (MoCA <19) | NR | NR | Atelectasis, prolonged mechanical ventilation, pleural effusion, pneumothorax, diaphragmatic dysfunction, pneumonia, pneumothorax, spirometry |
| Clement, 2011[11] | 1343 | 80-93 | 59.2 | Orthopedic | Inflammatory conditions | NR | NR | Transfusion, confusion, pneumonia, UTI, myocardial infarction, admission to a high dependency unit, re-admission, infection of prosthesis, DVT |
| Courtney-Brooks, 2012[12] | 37 | 65-95 | 100 | Gynecologic | History of Parksinon's disease, history of prior stroke, MMSE ≤18, either sinemet or donepezil as a current medication, an inability to walk 15ft or a known neurologic disorder affecting grip strength | 10 | 27 | Superficial and deep incisional surgical site infection (SSI), organ/space SSI, wound dehiscence, pneumonia, unplanned intubation for respiratory/cardiac failure, PE, ventilator support >48h, progressive renal insufficiency, acute renal failure requiring dialysis, UTI, stroke, coma>24h, peripheral nerve injury, cardiac arrest requiring CPR, myocardial infarction, bleeding requiring >4 units PRBCs within the first 72h after surgery, graft/prosthesis/flap failure, DVT or sepsis |
| Dales, 1993[13] | 15 | ≥75 vs. <50 | NR | Thoracic | NR | 8 | 53.3 | Atelectasis, pneumonia, empyema, hemothorax, PE, air leak, effusion, mechanical ventilation ≥72hrs, bronchopleural fistula, tension pneumothorax, pCO2>50mmHg at 24hrs, lobar gangrene |
| Dasgupta, 2009[14] | 125 | 70-92 | 58 | Orthopedic, vascular, abdominal, neurosurgical | Day surgical procedures, active cancer (defined as having surgery for a possible malignancy or receiving treatment for cancer, undecided as to whether they would have surgery, no working understanding of English, not cleared for surgery for unstable medical reasons, enrolled in RCT of new pharmacologic agents | 31 | 24.8 | Cardiac or pulmonary complications, delirium |
| Fukuse, 2005[15] | 120 | 60-84 | 40 | Thoracic | NR | 20 | 16.7 | Prolonged air leak, persistent air leak for >7 days requiring intercostal drainage, chylothorax, delirium, arrhythmias, pneumonia, atelectasis, pyelothorax |
| Gerson, 1985[16] | 155 | ≥65 | 51 | Abdominal, noncardiac thoracic | Atrial fibrillation, surgery cancelled | 23 | 23 | Cardiac death, ventricular tachycardia, ventricular fibrillation, myocardial infarction, congestive heart failure |
| Gerude, 2014[17] | 67 | 75-93 | 46.3 | ENT | Inability to walk; inability to answer questions due to hearing, cognitive, or speech deficits; impossibility of undergoing anthropometric measurement | 30 | 44.8 | Pneumonia, UTI, cerebrovascular disease, myocardial infarction, wound infection, wound dehiscence, wound bleeding, salivary fistula |
| Goto, 2007[18] | 720 | ≥60 | 68.2 | Cardiac | NR | 22 | 3 | Stroke |
| Green, 2012[19] | 159 | ≥60 | 50 | Cardiac | NR | NR | NR | In-hospital life-threatening and major bleeding events |
| Huisman, 2014[21] | 280 | 70-96 | 65 | Gastrointestinal, genitourinary, breast, ENT, other | NR | 135 | 48.2 | Clavien-Dindo classification |
| Javierre, 2012[22] | 874 | <70 vs. ≥70 | 37.4 | Cardiac | NR | 86 | 9.8 | Myocardial infarction |
| Kenig, 2015[23] | 75 | 65-93 | 44 | Abdominal | Perionteal carcinomatosis, only explorative laparoscopy/laparotomy | 38 | 51 | NR |
| Kim, 2013[24] | 141 | NR | 39.9 | General, urological, gynecological, thoracic, breast, ophthalmologic, ENT | NR | NR | NR | Delirium, pressure ulcers, pneumonia, UTIs |
| Kim, 2014[25] | 275 | ≥65 | 45.1 | Abdominal | Low risk of adverse outcome from surgery according to the ACC/AHA 2007 guidelines | 29 | 10.5 | Pneumonia, urinary tract infection, delirium, pulmonary embolus, unplanned ICU admission |
| Kim, 2016[26] | 197 | ≥69 | 51 | Vascular, orthopedic, urological, gynecological, ENT, other | NR | 30 | 15.2 | NR |
| Kothari, 2011[27] | 60 | ≥70 | 46.7 | Thoracic | Institutionalized patients that are not appropriate surgical candidate | 8 | 13 | Major complications |
| Kristjansson, 2010[28] | 182 | 70-94 | 57.1 | Colorectal | NR | NR | NR | Genitourinary, respiratory, cardiac, delirium, wound infection, intraabdominal abscess, anastomotic leakage, stroke, other |
| Lasithoiotakis, 2013[30] | 57 | 64.2-81.8 | 50.9 | Abdominal | Acute cholecystitis, jaundice, cholangitits, hydrops, empyema, or pancreatitis at the time of surgery | 13 | 22.7 | Fever, bleeding, reoperation, pancreatitis, subdiaphragmatic abscess, arrhythmia, atelectasis, pulmonary edema, UTI |
| Makary, 2010[33] | 594 | ≥65 | 41.9 | Intra-abdominal vs non-intra-abdominal | MMSE <18; history of Parkinson's disease or stroke; taking sinemet, donepezil, or antidepressants | NR | NR | NSQIP definitions |
| Papaioannou, 2005[3] | 47 | ≥60 | 36.2 | Orthopedic, urologic, vascular, gynecologic | Illiteracy, severe auditory or visual disturbances, central nervous system disorders, alcoholism or drug dependence, treatment with tranquillizers or antidepressants, Parkinson's disease, MMSE ≤23 | 13 | 27.6 | Wound disruptions, respiratory infections, PE, urinary retention, ileus, hyponatremia |
| Robinson, 2012[37] | 186 | ≥65 | 4.3 | Abdominal, cardiac, non-cardiac thoracic, vascular | Sensory impairment precluding delirium assessment, non-English speaking | 59 | 32 | Cardiac, respiratory, renal, neurologic, infection, sepsis, DVT, reoperation |
| Suh, 2014[39] | 60 | 70-85 | 100 | Gynecologic, general | NR | 18 | 30 | Wound dehiscence, delirium, hemorrhage, infection, vomiting, ileus, pulmonary problem, bowel perforation, fistula, urinary retention, electrolyte imbalance, glaucoma, mortality |
| Tan, 2012[42] | 83 | 75-93 | NR | Colorectal | Parkinsonism or taking levodopa or antidepressants | 22 | 26.5 | Life threatening or requiring significant deviation from standard management |
| van Venrooij, 2009[43] | 100 | ≥65 | 35 | Cardiac | Not able to keep a 3-day food record, not Dutch speaking | 25 | 25 | Organ failure, bleeding, infection |
| Williams, 2013[45] | 148 | ≥70 | 34 | Cardiac | Severe neuropsychiatric condition causing inability to cooperate with the study procedures; surgery cancelled | NR | NR | Stroke, renal failure, prolonged ventilation, deep sternal wound infection, reoperation |
| Zhang, 2012[46] | 100 | 60-85 | 35.9 | Noncardiac thoracic | NR | 67 | 67 | Atrial fibrillation, paroxysmal SVT, atrial/ventricular premature contraction, heart failure, sputum retention, hypoxemia, pulmonary air leak, atelectasis |

**Abbreviations:** ACC – American College of Cardiology; AHA – American Heart Association; ENT – otolaryngology; CPR – cardiopulmonary resuscitation; DVT – deep vein thrombosis; ICU – intensive care unit; MMSE – Mini–Mental State Examination; MoCA - Montreal Cognitive Assessment; NR – not reported; NSQIP – National Surgical Quality Improvement Program; PE – pulmonary embolism; PRBCs – packed red blood cells; RCT – randomized controlled trial; SSI - surgical site infection; SVT - Supraventricular tachycardia; UTI – urinary tract infection

# Appendix 7. Forest plots of study-level and pooled effect estimates for prognostic factors associated with postoperative complications among older adults undergoing elective surgery


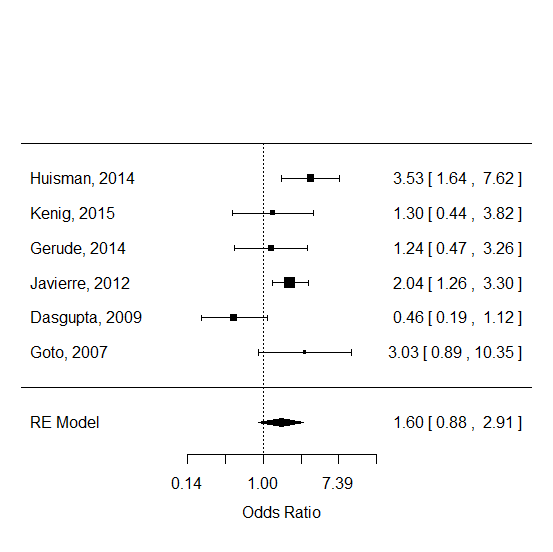


**Figure 1.** Forest plot of the study-level effect measures and the summary effect measure for the odds of postoperative complications in a male patient (I^2^=66.24%).


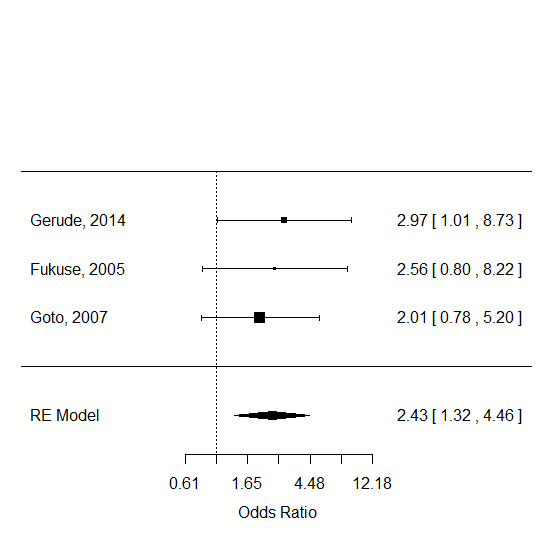


**Figure 2.** Forest plot of the study-level effect measures and the summary effect measure for the odds of postoperative complications in a patient who smokes cigarettes (I^2^=0%).


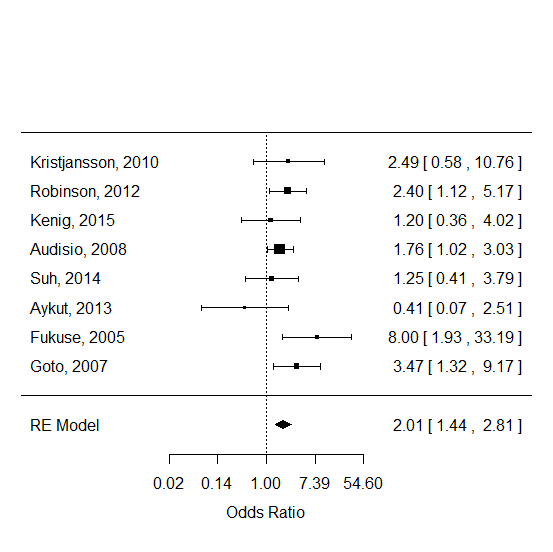


**Figure 3.** Forest plot of the study-level effect measures and the summary effect measure for the odds of postoperative complications in a patient with cognitive impairment (I^2^=0%).


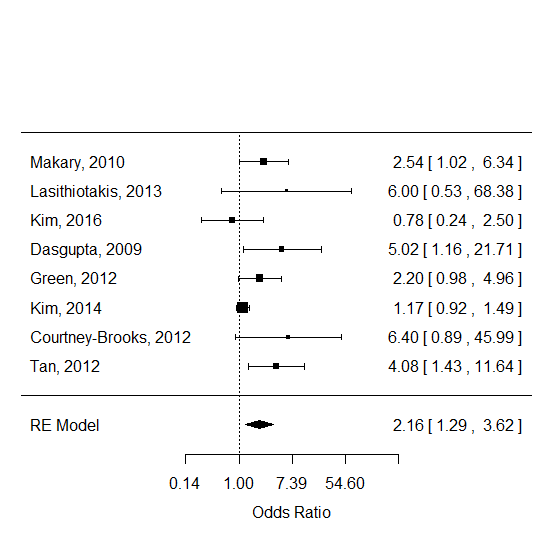


**Figure 4a.** Forest plot of the study-level effect measures and the summary effect measure for the odds of postoperative complications in a patient with frailty (I^2^=54.69%).


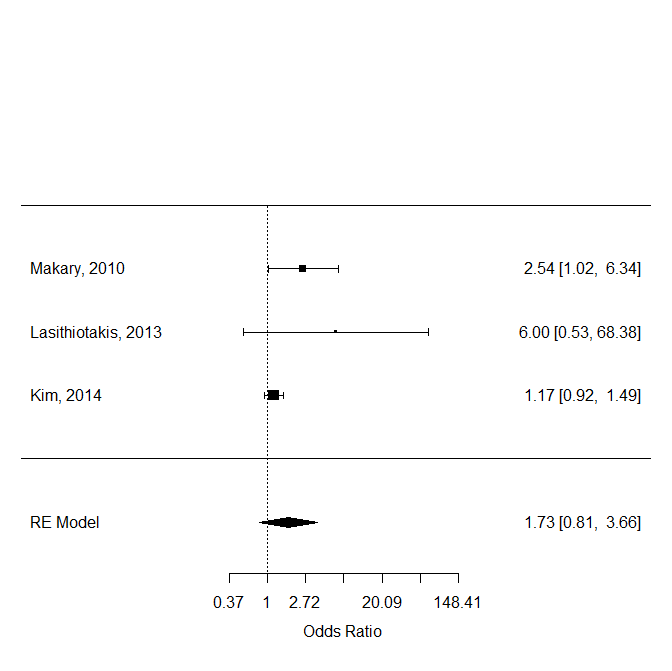


**Figure 4b.** Forest plot of the study-level effect measures and the summary effect measure for the odds of postoperative complications in a patient with frailty undergoing abdominal surgery (I^2^=53.36%).


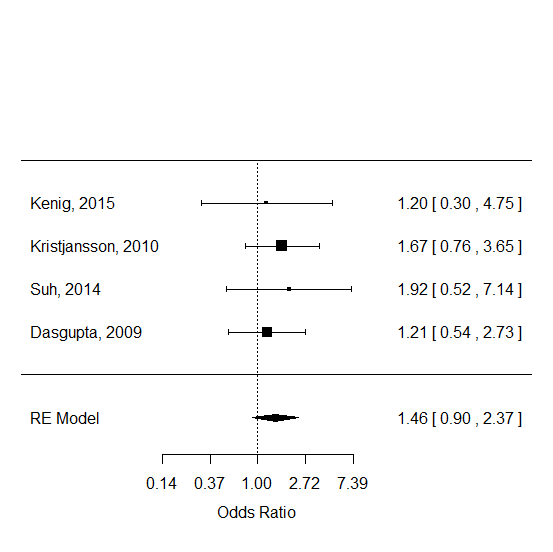


**Figure 5.** Forest plot of the study-level effect measures and the summary effect measure for the odds of postoperative complications in a patient with polypharmacy (I^2^=0%).


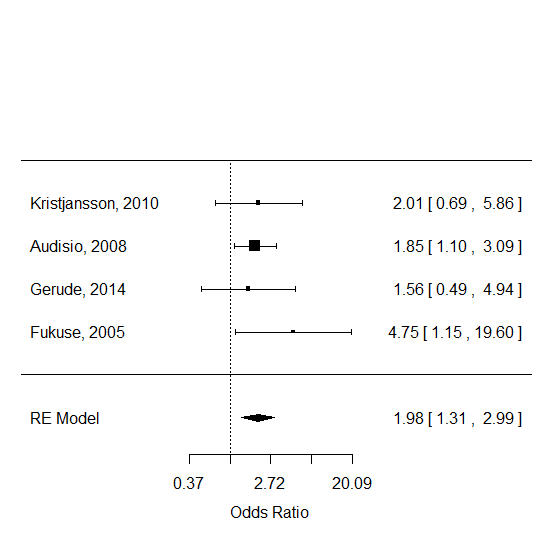


**Figure 6.** Forest plot of the study-level effect measures and the summary effect measure for the odds of postoperative complications in a patient with impairment in activities of daily living (I^2^=0%).


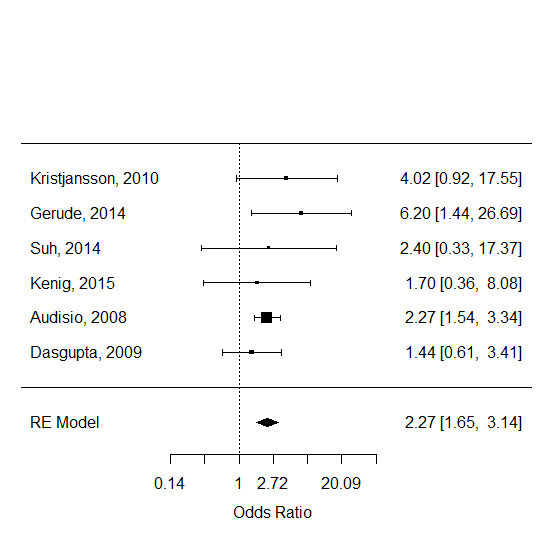


**Figure 7.** Forest plot of the study-level effect measures and the summary effect measure for the odds of postoperative complications in a patient with impairment in instrumental activities of daily living (I^2^=0%).


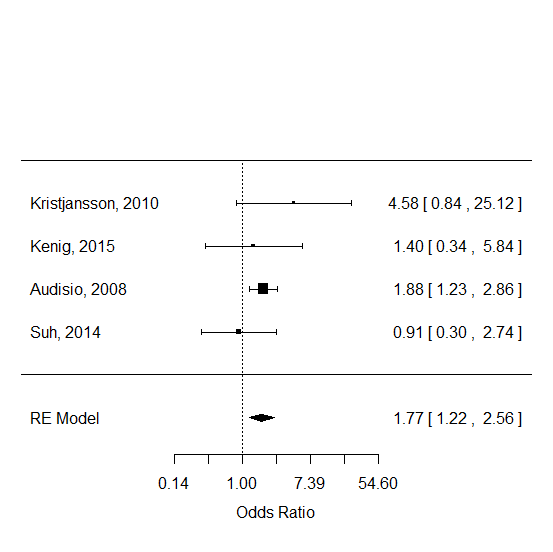


**Figure 8.** Forest plot of the study-level effect measures and the summary effect measure for the odds of postoperative complications in a patient with depressive symptoms as measured by the Geriatric Depression Screen (GDS) (I^2^=0%).


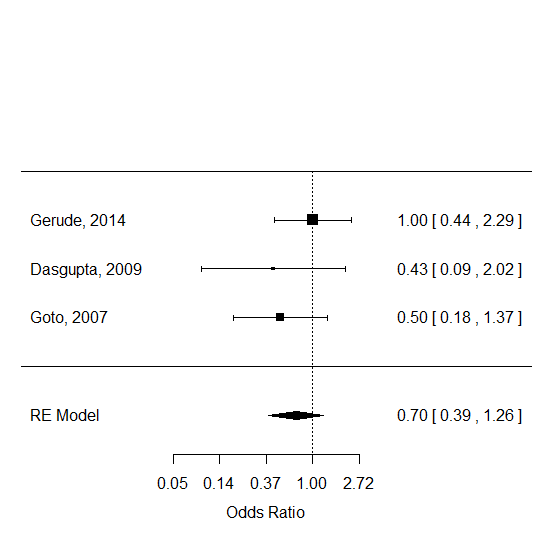


**Figure 9.** Forest plot of the study-level effect measures and the summary effect measure for the odds of postoperative complications in a patient with diabetes mellitus (I^2^=0%).


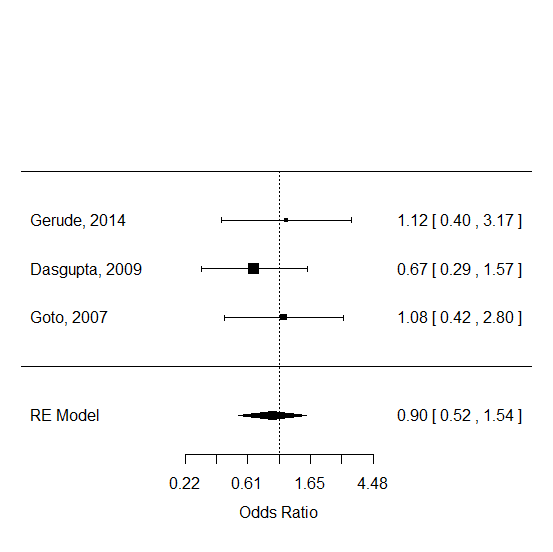


**Figure 10.** Forest plot of the study-level effect measures and the summary effect measure for the odds of postoperative complications in a patient with hypertension (I^2^=0%).


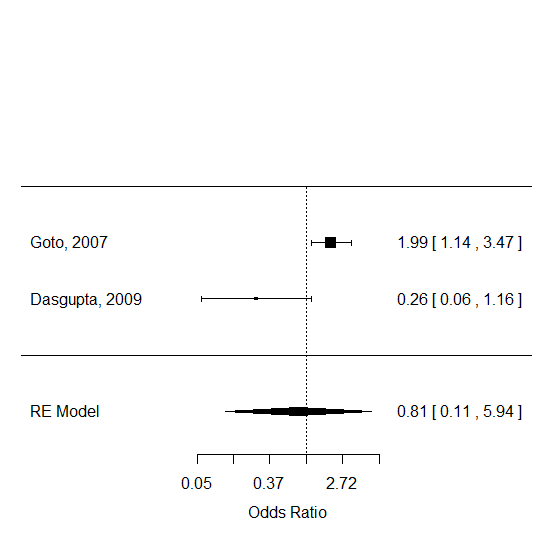


**Figure 11.** Forest plot of the study-level effect measures and the summary effect measure for the odds of postoperative complications in a patient with a history of cerebrovascular disease (I^2^=83.39%).


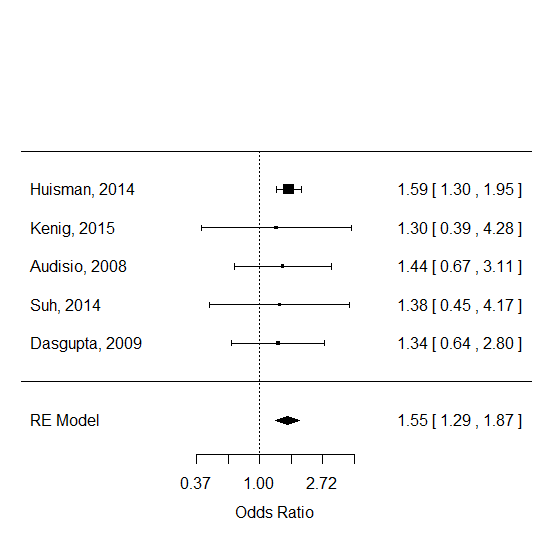


**Figure 12a.** Forest plot of the study-level effect measures and the summary effect measure for the odds of postoperative complications in a patient with a greater comorbidity score (I^2^=0%).


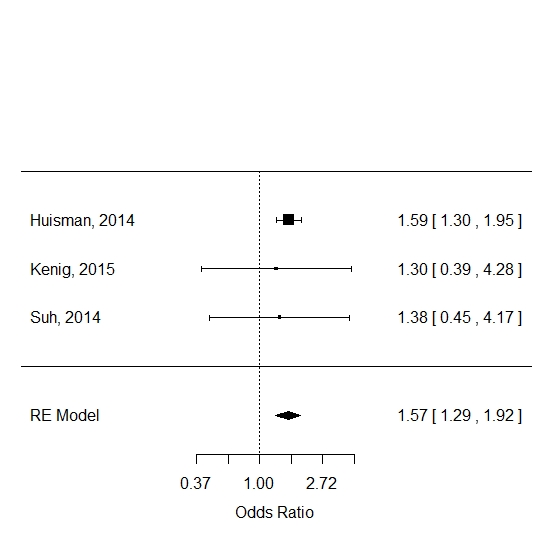


**Figure 12b.** Forest plot of the study-level effect measures and the summary effect measure for the odds of postoperative complications in a patient with a greater Charlson comorbidity score (I^2^=0%).


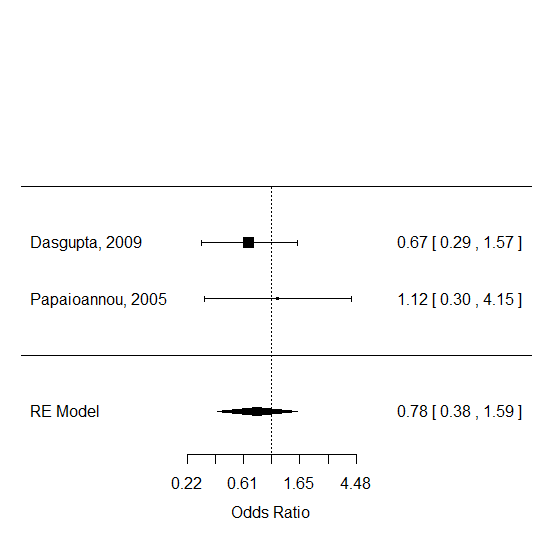


**Figure 13.** Forest plot of the study-level effect measures and the summary effect measure for the odds of postoperative complications in a patient undergoing elective surgery under general anesthesia (I^2^=0%).


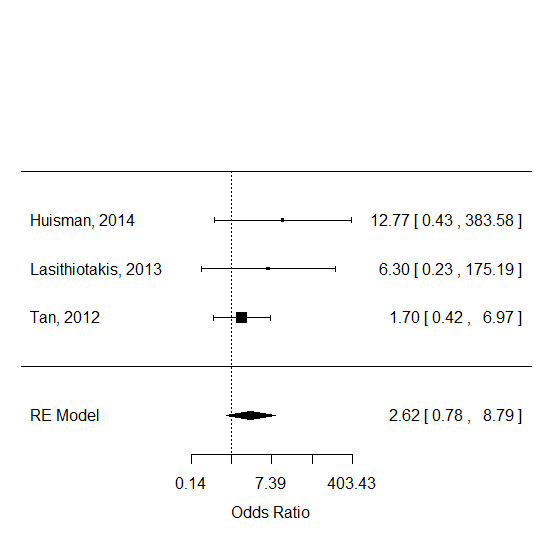


**Figure 14.** Forest plot of the study-level effect measures and the summary effect measure for the odds of postoperative complications in a patient with ASA Score ≥3 (I^2^=0%).


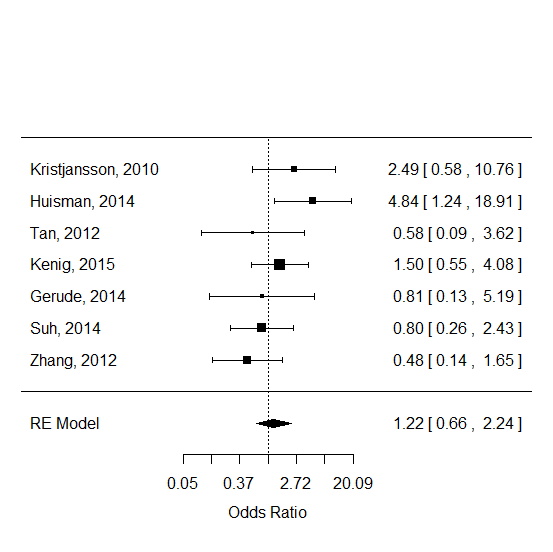


**Figure 15.** Forest plot of the study-level effect measures and the summary effect measure for the odds of postoperative complications in a patient with malnutrition (I^2^=31.02%).


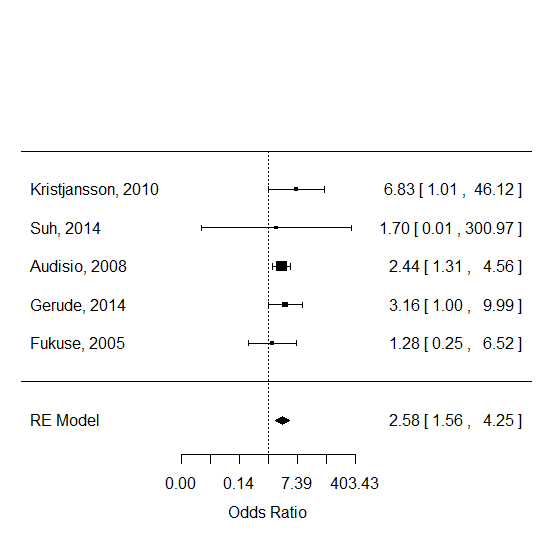


**Figure 16.** Forest plot of the study-level effect measures and the summary effect measure for the odds of postoperative complications in a patient with poor performance status (I^2^=0%).


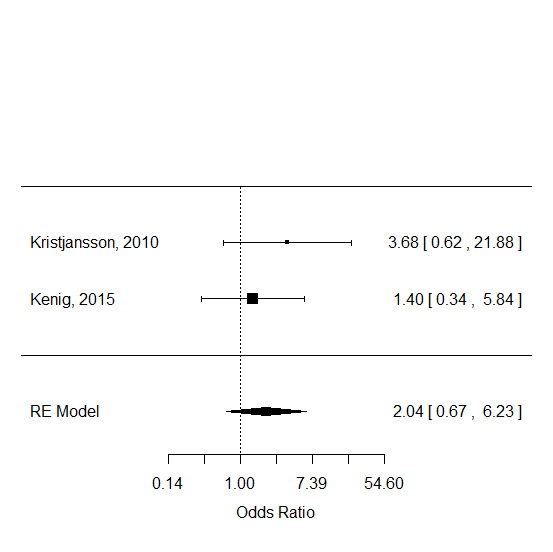


**Figure 17.** Forest plot of the study-level effect measures and the summary effect measure for the odds of postoperative complications in a patient with depression (I^2^=0%).


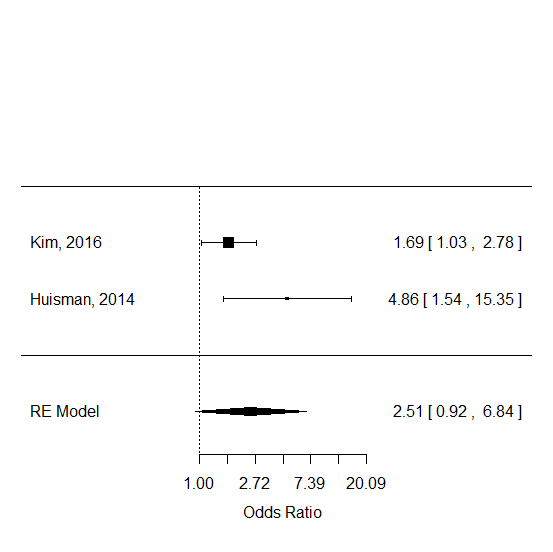


**Figure 18.** Forest plot of the study-level effect measures and the summary effect measure for the odds of postoperative complications in a patient with poor mobility (I^2^=63.37%).


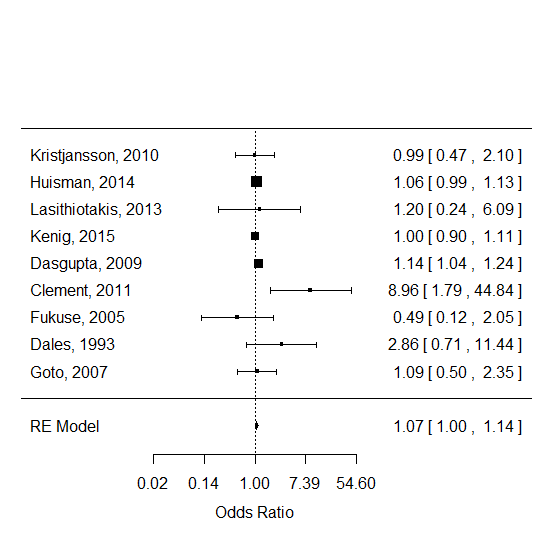


**Figure 19.** Forest plot of the study-level effect measures and the summary effect measure for the odds of postoperative complications in a patient with older age (I^2^=17.96%).

# Appendix 8. Forest plots of the study-level and pooled effect estimates of the prognostic factors associated with postoperative mortality among older adults undergoing elective surgery


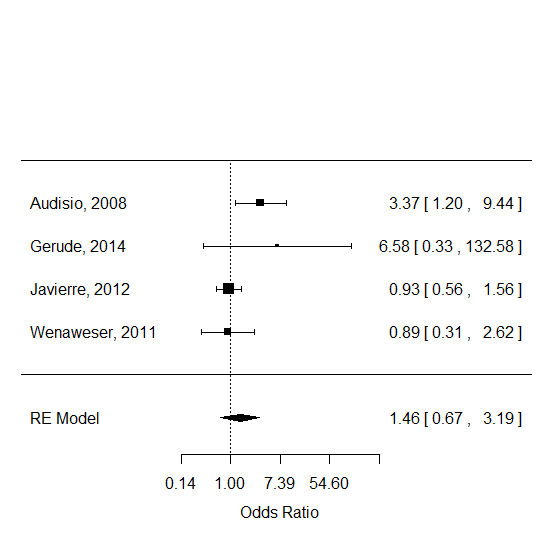


**Figure 20.** Forest plot of the study-level effect measures and the summary effect measure for the odds of postoperative mortality in a male patient (I^2^=53.92%).


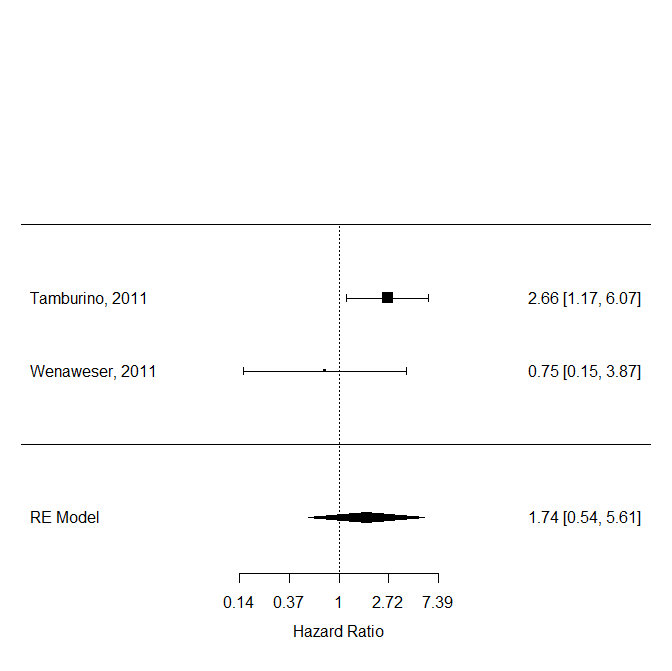


**Figure 21.** Forest plot of the study-level effect measures and the summary effect measure for the hazard ratio associated with postoperative mortality in a patient with diabetes mellitus (I^2^=45.26%).


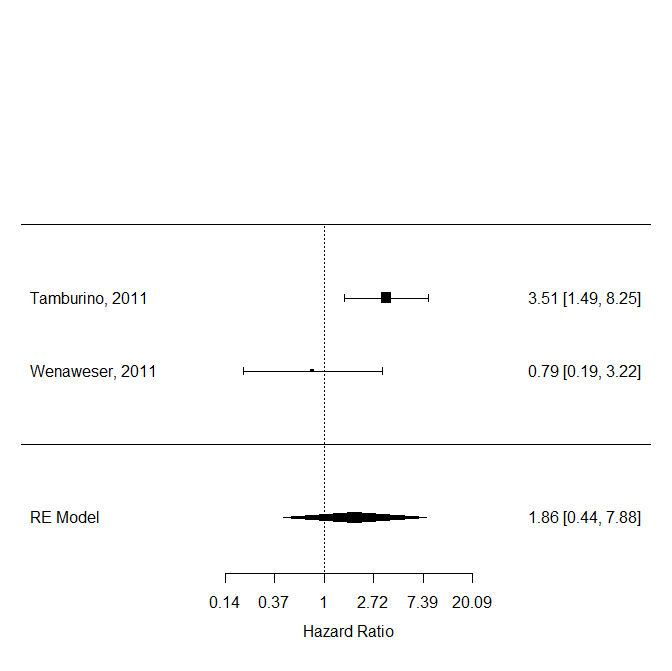


**Figure 22.** Forest plot of the study-level effect measures and the summary effect measure for the hazard ratio associated with postoperative mortality in a patient with heart failure (I^2^=68.34%).

# Appendix 9. Forest plot of study-level and pooled effect estimates for prognostic factors associated with prolonged hospitalization among older adults undergoing elective surgery


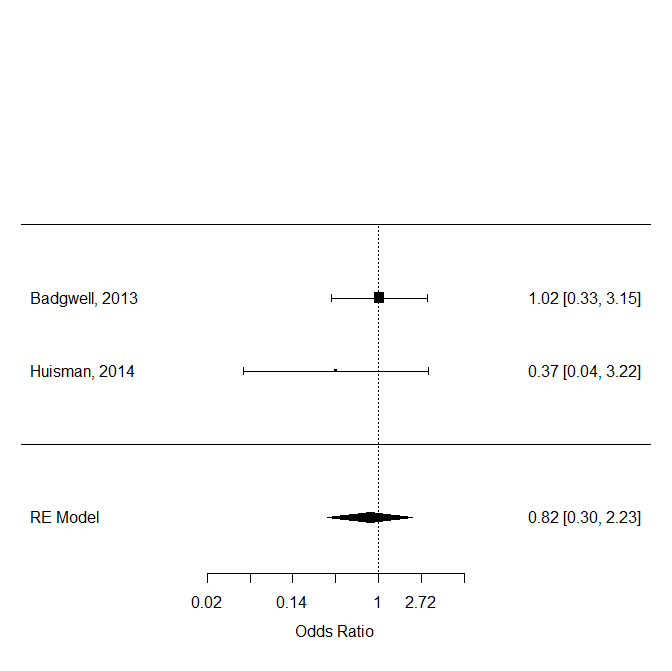


**Figure 23.** Forest plot of the study-level effect measures and the summary effect measure for the odds of prolonged hospitalization associated with elective surgery in a patient with higher ASA score (I^2^=0%)

# Appendix 10. Forest plot of study-level and pooled effect estimates for prognostic factors associated with destination at discharge from hospital among older adults undergoing elective surgery


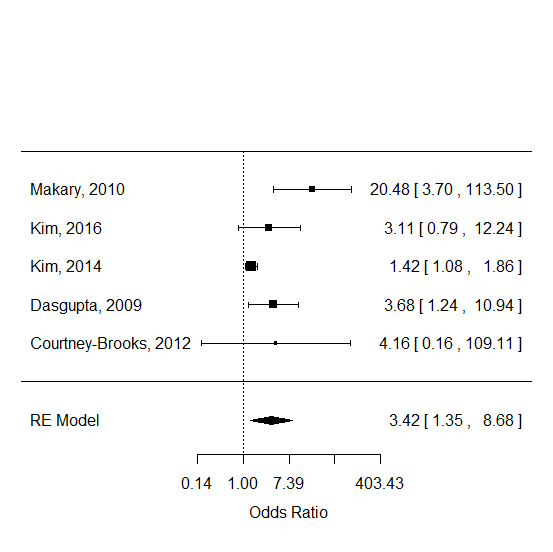


**Figure 24.** Forest plot of the study-level effect measures and the summary effect measure for the odds of not being discharged home following hospitalization for elective surgery in a patient with frailty (I^2^=67.46%).

# References

1. Sanchez-Meca J, Marin-Martinez F, Chacon-Moscoso S. Effect-size indices for dichotomized outcomes in meta-analysis. Psychol Methods. 2003;8(4):448-467.

2. Cochrane Handbook for Systematic Reviews of Interventions Version 5.1.0 [updated March 2011]. The Cochrane Collaboration, 2011. Available from [www.handbook.cochrane.org](http://www.handbook.cochrane.org).

3. Papaioannou A, Fraidakis O, Michaloudis D, Balalis C, Askitopoulou H. The impact of the type of anaesthesia on cognitive status and. European Journal of Anesthesiology. 2005;22:492-499.

4. Schmidt M, Eckardt R, Scholtz K, Neuner B, Von Dossow-Hanfstingl V, Sehouli J et al. Patient empowerment improved Perioperative quality of care in cancer patients aged > 65 Years - a randomized controlled trial. PLoS ONE. 2015;10:1-22.

5. Amemiya T, Oda K, Ando M, Kitagawa Y, Shimada H, Kuroiwa K et al. Activities of Daily Living and Quality of Life of Elderly Patients After Elective Surgery for Gastric and Colorectal Cancers. Ann Surg. 2007;246:222-228.

6. Audisio RA, Pope D, Gennari R, Maffezzini M, Hoekstra HJ, Mobarak D et al. Shall we operate? Preoperative assessment in elderly cancer patients (PACE) can help. A SIOG surgical task force prospective study. Critical Reviews in Oncology/Hematology. 2008;65:156-163.

7. Aykut K, Albayrak G, Guzeloglu M, Baysak A, Hazan E. Preoperative mild cognitive dysfunction predicts pulmonary complications after coronary artery bypass graft surgery. J Cardiothorac Vasc Anesth. 2013;27(6):1267-1270.

8. Badgwell B, Stanley J, Chang GJ, Katz MH, Lin HY, Ning J et al. Comprehensive geriatric assessment of risk factors associated with adverse outcomes and resource utilization in cancer patients undergoing abdominal surgery. J Surg Oncol. 2013;108(3):182-186.

9. Betomvuko P, Michaux I, Gabriel L, Bihin B, Gourdin M, De Saint Hubert M: Gait speed as predictor of outcomes of elective cardiac surgery in older patients. In: European Union Geriatric Medicine Society (EUGMS) Congress: 2015; Oslo, Norway: European Geriatric Medicine; 2015: S147.

10. Blakoe M, Greve H: Frailty - preoperative assessment and implications for nursing practice following heart surgery. In: EuroHeartCare: 2015; Dubrovnik, Croatia: European Journal of Cardiovascular Nursing; 2015: 44.

11. Clement ND, MacDonald D, Howie CR, Biant LC. The outcome of primary total hip and knee arthroplasty in patients aged 80 years or more. J Bone Joint Surg Br. 2011;93(9):1265-1270.

12. Courtney-Brooks M, Tellawi AR, Scalici J, Duska LR, Jazaeri AA, Modesitt SC et al. Frailty: An outcome predictor for elderly gynecologic oncology patients. Gynecologic Oncology. 2012;126:20-24.

13. Dales RE, Dionne G, Leech JA, Lunau M, Schweitzer I. Preoperative prediction of pulmonary complications following thoracic surgery. Chest. 1993;104(1):155-159.

14. Dasgupta M, Rolfson DB, Stolee P, Borrie MJ, Speechley M. Frailty is associated with postoperative complications in older adults with medical problems. Archives of Gerontology and Geriatrics. 2009;48:78-83.

15. Fukuse T, Satoda N, Hijiya K, Fujinaga T. Importance of a comprehensive geriatric assessment in prediction of complications following thoracic surgery in elderly patients. Chest. 2005;127:886-891.

16. Gerson MC, Hurst JM, Hertzberg VS, Doogan PA, Cochran MB, Lim SP et al. Cardiac prognosis in noncardiac geriatric surgery. Ann Intern Med. 1985;103(6 ( Pt 1)):832-837.

17. Gerude MF, Dias FL, de Farias TP, Albuquerque Sousa B, Thuler LC. Predictors of postoperative complications, prolonged length of hospital stay, and short-term mortality in elderly patients with malignant head and neck neoplasm. ORL J Otorhinolaryngol Relat Spec. 2014;76(3):153-164.

18. Goto T, Baba T, Ito A, Maekawa K, Koshiji T. Gender differences in stroke risk among the elderly after coronary artery surgery. Anesth Analg. 2007;104(5):1016-1022, tables of contents.

19. Green P, Woglom AE, Genereux P, Daneault B, Paradis JM, Schnell S et al. The impact of frailty status on survival after transcatheter aortic valve replacement in older adults with severe aortic stenosis: A single-center experience. JACC: Cardiovascular Interventions. 2012;5:974-981.

20. Hoogerduijn JG, de Rooij SE, Grobbee DE, Schuurmans MJ. Predicting functional decline in older patients undergoing cardiac surgery. Age Ageing. 2014;43(2):218-221.

21. Huisman MG, Van Leeuwen BL, Ugolini G, Montroni I, Spiliotis J, Stabilini C et al. "Timed Up & Go": A screening tool for predicting 30-day morbidity in onco-geriatric surgical patients? A multicenter cohort study. PLoS ONE. 2014;9.

22. Javierre C, Ricart A, Manez R, Farrero E, Carrio ML, Rodriguez-Castro D et al. Age and sex differences in perioperative myocardial infarction after cardiac surgery. Interact Cardiovasc Thorac Surg. 2012;15(1):28-32.

23. Kenig J, Olszewska U, Zychiewicz B, Barczynski M, Mitu??-Kenig M. Cumulative deficit model of geriatric assessment to predict the postoperative outcomes of older patients with solid abdominal cancer. Journal of Geriatric Oncology. 2015;6:370-379.

24. Kim KI, Park KH, Koo KH, Han HS, Kim CH. Comprehensive geriatric assessment can predict postoperative morbidity and mortality in elderly patients undergoing elective surgery. Arch Gerontol Geriatr. 2013;56(3):507-512.

25. Kim S-W, Han H-S, Jung H-W, Kim K-I, Hwang DW, Kang S-B et al. Multidimensional frailty score for the prediction of postoperative mortality risk. JAMA surgery. 2014;149:633-640.

26. Kim S, Marsh AP, Rustowicz L, Roach C, Leng XI, Kritchevsky SB et al. Self-reported Mobility in Older Patients Predicts Early Postoperative Outcomes after Elective Noncardiac Surgery. Anesthesiology. 2016;124:815-825.

27. Kothari A, Phillips S, Bretl T, Block K, Weigel T. Components of geriatric assessments predict thoracic surgery outcomes. J Surg Res. 2011;166(1):5-13.

28. Kristjansson SR, Jordh??y MS, Nesbakken A, Skovlund E, Bakka A, Johannessen HO et al. Which elements of a comprehensive geriatric assessment (CGA) predict post-operative complications and early mortality after colorectal cancer surgery? Journal of Geriatric Oncology. 2010;1:57-65.

29. Kwon S, Symons R, Yukawa M, Dasher N, Legner V, Flum DR. Evaluating the association of preoperative functional status and postoperative functional decline in older patients undergoing major surgery. Am Surg. 2012;78(12):1336-1344.

30. Lasithiotakis K, Petrakis J, Venianaki M, Georgiades G, Koutsomanolis D, Andreou A et al. Frailty predicts outcome of elective laparoscopic cholecystectomy in geriatric patients. Surg Endosc. 2013;27(4):1144-1150.

31. Lawrence VA, Hazuda HP, Cornell JE, Pederson T, Bradshaw PT, Mulrow CD et al. Functional Independence after Major Abdominal Surgery in the Elderly. J Am Coll Surg. 2004;199:762-772.

32. Legner VJ, Doerner D, Reilly DF, Mccormick WC. Risk Factors for Nursing Home Placement following Major Nonemergent Surgery. The American Journal of Medicine. 2004;117:82-86.

33. Makary MA, Segev DL, Pronovost PJ, Syin D, Bandeen-Roche K, Patel P et al. Frailty as a predictor of surgical outcomes in older patients. J Am Coll Surg. 2010;210(6):901-908.

34. Min L, Mazzurco L, Gure TR, Cigolle CT, Lee P, Bloem C et al. Longitudinal functional recovery after geriatric cardiac surgery. J Surg Res. 2015;194(1):25-33.

35. Pirracchio R, Resche-Rigon M, Bresson D, Basta B, Welschbillig S, Heyer L et al. One-year outcome after neurosurgery for intracranial tumor in elderly patients. J Neurosurg Anesthesiol. 2010;22(4):342-346.

36. Reinöhl J, Kaier K, Gutmann A, Sorg S, von Zur Mühlen C, Siepe M et al. In-hospital resource utilization in surgical and transcatheter aortic valve replacement. BMC cardiovascular disorders. 2015;15:132.

37. Robinson TN, Wu DS, Pointer LF, Dunn CL, Moss M. Preoperative cognitive dysfunction is related to adverse postoperative outcomes in the elderly. J Am Coll Surg. 2012;215(1):12-17.

38. Rogers MP, Liang MH, Daltroy LH, Eaton H, Peteet J, Wright E et al. Delirium after elective orthopedic surgery: risk factors and natural history. Int J Psychiatry Med. 1989;19(2):109-121.

39. Suh DH, Kim JW, Kim HS, Chung HH, Park NH, Song YS. Pre- and intra-operative variables associated with surgical complications in elderly patients with gynecologic cancer: the clinical value of comprehensive geriatric assessment. J Geriatr Oncol. 2014;5(3):315-322.

40. Sundermann SH, Dademasch A, Seifert B, Biefer HRC, Emmert MY, Walther T et al. Frailty is a predictor of short- and mid-term mortality after elective cardiac surgery independently of age. Interactive Cardiovascular and Thoracic Surgery. 2014;18:580-585.

41. Tamburino C, Capodanno D, Ramondo A, Petronio AS, Ettori F, Santoro G et al. Incidence and predictors of early and late mortality after transcatheter aortic valve implantation in 663 patients with severe aortic stenosis. Circulation. 2011;123(3):299-308.

42. Tan KY, Kawamura YJ, Tokomitsu A, Tang T. Assessment for frailty is useful for predicting morbidity in elderly patients undergoing colorectal cancer resection whose comorbidities are already optimized. The American Journal of Surgery. 2012;204:139-143.

43. van Venrooij LM, van Leeuwen PA, de Vos R, Borgmeijer-Hoelen MM, de Mol BA. Preoperative protein and energy intake and postoperative complications in well-nourished, non-hospitalized elderly cardiac surgery patients. Clin Nutr. 2009;28(2):117-121.

44. Wenaweser P, Pilgrim T, Roth N, Kadner A, Stortecky S, Kalesan B et al. Clinical outcome and predictors for adverse events after transcatheter aortic valve implantation with the use of different devices and access routes. Am Heart J. 2011;161(6):1114-1124.

45. Williams JB, Alexander KP, Morin JF, Langlois Y, Noiseux N, Perrault LP et al. Preoperative anxiety as a predictor of mortality and major morbidity in patients aged >70 years undergoing cardiac surgery. Am J Cardiol. 2013;111(1):137-142.

46. Zhang L, Wang C, Sha SY, Kwauk S, Miller AR, Xie MS et al. Mini-nutrition assessment, malnutrition, and postoperative complications in elderly Chinese patients with lung cancer. J buon. 2012;17(2):323-326.
